# Extracorporeal Hemadsorption *versus* Glucocorticoids during Cardiopulmonary Bypass: A Prospective, Randomized, Controlled Trial

**DOI:** 10.1155/2020/7834173

**Published:** 2020-03-27

**Authors:** Gordana Taleska Stupica, Maja Sostaric, Marija Bozhinovska, Lea Rupert, Zoran Bosnic, Ales Jerin, Alojz Ihan, Tomislav Klokocovnik, Matej Podbregar

**Affiliations:** ^1^Clinical Department of Anaesthesiology and Perioperative Intensive Therapy, University Medical Centre Ljubljana, Ljubljana, Slovenia; ^2^University of Ljubljana, Faculty of Medicine, Ljubljana, Slovenia; ^3^University of Ljubljana, Faculty of Computer and Information Science, Ljubljana, Slovenia; ^4^Institute of Clinical Chemistry and Biochemistry, University Medical Centre Ljubljana, Ljubljana, Slovenia; ^5^University of Ljubljana, Faculty of Medicine, Institute of Microbiology and Immunology, Ljubljana, Slovenia; ^6^Clinical Department of Cardio-Vascular Surgery, University Medical Centre Ljubljana, Ljubljana, Slovenia; ^7^Department of Internal Intensive Medicine, General and Teaching Hospital, Celje, Slovenia

## Abstract

Extracorporeal hemadsorption may reduce inflammatory reaction in cardiopulmonary bypass (CPB) surgery. Glucocorticoids have been used during open-heart surgery for alleviation of systemic inflammation after CPB. We compared intraoperative hemadsorption and methylprednisolone, with usual care, during complex cardiac surgery on CPB, for inflammatory responses, hemodynamics, and perioperative course. Seventy-six patients with prolonged CPB were recruited and randomized, with 60 included in final analysis. Allocation was into three groups: Methylprednisolone (*n* = 20), Cytosorb (*n* = 20), and Control group (usual care, *n* = 20). Proinflammatory (TNF-*α*, IL-1*β*, IL-6, and IL-8) and anti-inflammatory (IL-10) cytokines which complement C5a, CD64, and CD163 expression by immune cells were analyzed within the first five postoperative days, in addition to hemodynamic and clinical outcome parameters. Methylprednisolone group, compared to Cytosorb and Control had significantly lower levels of TNF-*α* (until the end of surgery, *p* < 0.001), IL-6 (until 48 h after surgery, *p* < 0.001), and IL-8 (until 24 h after surgery, *p* < 0.016). CD64 expression on monocytes was the highest in the Cytosorb group and lasted until the 5^th^ postoperative day (*p* < 0.016). IL-10 concentration (until the end of surgery) and CD163 expression on monocytes (until 48 h after surgery) were the highest in the Methylprednisolone group (*p* < 0.016, for all measurements between three groups). No differences between groups in the cardiac index or clinical outcome parameters were found. Methylprednisolone more effectively ameliorates inflammatory responses after CPB surgery compared to hemadsorption and usual care. Hemadsorption compared with usual care causes higher prolonged expression of CD64 on monocytes but short lasting expression of CD163 on granulocytes. Hemadsorption with CytoSorb® was safe and well tolerated. This trial is registered with clinicaltrials.gov (NCT02666703).

## 1. Introduction

Cardiopulmonary bypass (CPB) is almost unavoidable for most open-heart surgical procedures although undesirable complex inflammatory response known as systemic inflammatory response syndrome (SIRS) is associated with its use. Balance of proinflammatory and anti-inflammatory mediators determines this inflammatory response and thus the clinical outcome [1–3].

Many efforts during the past years have been focused on therapeutic interventions to reduce inflammatory reactions during CPB [4–6]. Among pharmacological strategies, prophylaxis with glucocorticoids has been used during open-heart surgery for more than 30 years. However, experimental and clinical studies produced contradictory evidence regarding steroid treatment benefits, particularly those related with patients' clinical outcome [7–13].

Extracorporeal cytokine adsorption, known as hemadsorption, uses biocompatible highly porous polymer cartridges and is generally indicated when cytokines might be elevated. There appears not to be any serious unwanted side effects associated with this device, except for a small reduction in the number of platelets [14–20].

Encouraged by promising data from the use of hemadsorption in septic patients, recently the use of this therapeutic method has been expanded to cardiac surgery for reducing systemic inflammation after CPB. However, the first published data, both retrospective and prospective, have been confusing, with reports of both important alleviation [21, 22] of inflammation and no significant benefits [23].

The aim of this trial was to compare the effects of hemadsorption (CytoSorb® cartridge) during prolonged CPB with a median dose of glucocorticoid and with usual care, for the control of inflammatory responses after complex cardiac surgery and the impact of these therapeutic strategies on patient outcome.

## 2. Methods

This prospective, randomized, blinded, interventional, single-centre controlled clinical trial was carried out at the Clinical Department of Anaesthesiology and Perioperative Intensive Therapy, Division of Cardiovascular Anaesthesia, and the Clinical Department of Cardiovascular Surgery at the University Medical Centre in Ljubljana (Slovenia) between February 2016 and December 2017. Approval for this study was obtained from the National Medical Ethics Committee (Affiliation: Ministry of Health of Republic of Slovenia; approval number: 118/02/15) and was accompanied by a written signed informed consent of each patient. The study was conducted in accordance with the Helsinki Declaration and registered at ClinicalTrials.gov (NCT02666703) before patient recruitment started.

Of 76 patients enrolled, 60 patients assigned to elective complex cardiac surgery with prolonged duration of CPB (>90 min) were eligible for inclusion in final analysis. These patients were randomized into three study groups: Methylprednisolone, MP (*n* = 20; 1 g of methylprednisolone added in the CPB priming solution), Cytosorb, CS (*n* = 20; CytoSorb® cartridge, Cytosorbents Europe GmbH, Germany, installed in the CPB circuit), and Control, CO (*n* = 20, usual care, neither methylprednisolone nor CytoSorb® during CPB).

Randomization was carried out by one of the members of the study team a day before surgery and achieved by using identical sealed envelopes, whereby each patient selected an envelope that assigned him/her to one of three treatment groups. Randomization allocation numbers were generated by the Research Randomizer (https://www.randomizer.org/).

Patients, ICU and ward personnel, and laboratory staff who participated in the trial were “*blinded*” for assigned treatment throughout the duration of the study. Exception from being blinded was for personnel in the operating theatre, who, on the other hand, were not included in data collection and analysis.

Based on previous literature [23], power of study calculation was based on assumption that a change in the mean difference of one standard deviation would suffice as a clinically relevant effect for a two-side test. To achieve 80% statistical power with a significance level (*α*) of 5%, the ClinCalc sample size calculator defined the need for 17 patients per group, which indicated 51 patients across the three treatment groups. Considering an estimated 15% drop-out rate, 60 patients were included in final analysis to avoid risk of low power.

### 2.1. Inclusion and Exclusion Criteria

This study included patients >18 years old who were admitted for elective complex cardiac surgery with an expected CPB duration of >90 min. The surgery thus included combined valve and coronary bypass grafting surgery, concomitant surgery of two or more valves, surgery of ascending aorta and aortic arch, and reoperations of the same types.

Exclusion criteria included refusal to participate in the study; age <18 years; pregnant women; emergency procedures; heart transplantation; implantation of the left ventricular assist device, right ventricular assist device, or total artificial heart; treatment with chemo/immunosuppressive therapy; treatment with antileukocyte drugs or TNF-*α* blockers; immunocompromised patients (e.g., with AIDS); leucopenia (<4.0 × 10^9^ cells·L^−1^); clinical and/or laboratory signs of infection (i.e., C-reactive protein (CRP), >2 mg·dL^−1^ [20 mg·L^−1^]; procalcitonin, >0.5 *μ*g·L^−1^; leukocytes, >10.0 × 10^9^ cells·L^−1^); serum creatinine >2 mg·dL^−1^ (176 *μ*mol·L^−1^); bilirubin >2 mg·dL^−1^ (34.2 *μ*mol·L^−1^); history of stroke; malnourished patients; body mass index <18 kg·(m^2^)^−1^.

### 2.2. Procedure

After preoperative assessment and premedication with benzodiazepines, the patients were operated on under general anaesthesia. Before induction of general anaesthesia, each patient had an arterial cannula inserted in their radial artery, for the hemodynamic measurements (FloTrac™ System, Edwards Lifesciences; USA) and for drawing of blood samples for analysis. Induction of general anaesthesia was intravenous, using fentanyl 5–10 *μ*g/kg, propofol 1-2 mg/kg, and rocuronium bromide 0.6 mg/kg, for tracheal intubation. Patients were ventilated with volume-controlled ventilation with a tidal volume (Vt) of 6–8 mL/kg ideal body weight, with the intention to maintain normocarbia. After induction of general anaesthesia, a central venous catheter was inserted (PreSep Central Venous Oximetry catheter; Edwards Lifesciences, USA) for hemodynamic measurements and a high-flow device (AVA; Edwards Lifesciences, USA) for fluid replacement therapy (both in the jugular veins). Total intravenous anaesthesia was maintained throughout the whole surgical procedure using continuous infusions of propofol (dose according to the measurement of depth of anaesthesia with the bispectral index) and remifentanil (0.3–0.5 *μ*g/kg/min).

Standard and extended hemodynamic monitoring was maintained intraoperatively, including electrocardiogram, invasive arterial blood pressure, central venous pressure, cardiac index, systemic vascular resistance index, and central venous oxygen saturation (EV 1000 Platform; Edwards Lifesciences, USA). We also monitored hemoglobin oxygen saturation, capnography (end tidal CO_2_), cerebral oxygen saturation (near infrared spectroscopy; INVOS Cerebral/Somatic Oximeter, Medtronic, USA), depth of anaesthesia (bispectral index; Brain Monitoring System, Medtronic, USA), body temperature, urine output, and acid/base status with blood gas analysis. The type and duration of surgery were recorded along with duration of CPB and aortic cross-clamp, as well as blood loss, quantity of crystalloids, colloids, blood components, procoagulant factors administered, inotropic and/or vasoactive drug consumption, and insulin used. During each surgical procedure, transoesophageal echocardiography was used to determine global cardiac function, regional wall motion abnormalities, valvular function, volume status, and deaeration before unclamping of the aorta.

The CPB used was standard and mildly hypothermic (32–34°C), with a nonpulsatile flow of 2.2 to 2.4 L·m^2, −1^ body surface area. Two types of oxygenators were used: Inspire system (Sorin, Milan, Italy), coated with a phosphoryl choline inert surface, and Fusion system (Medtronic, Minneapolis, USA), coated with a hydrophilic polymer. Priming solution included 1200 mL lactated ringer, 250 mL 20% mannitol, and 100 mg heparin. For the Methylprednisolone group, methylprednisolone 1 g was added to the priming solution, which is used to fill CPB machine. For the Cytosorb group, the CytoSorb® cartridge was installed on CPB machine in a parallel circuit to body circulation. Flow through the filter was derived from the arterial line for CPB and was driven by an additional roller pump at 400 mL/min, to the venous reservoir, in order to provide equal conditions during CPB for each study patient. For the Control group, neither methylprednisolone nor CytoSorb® filter was used with CPB.

Norepinephrine was used as the main vasoactive drug, and its administration was MAP guided, aiming for 70–75 mmHg throughout the perioperative course. Blood transfusion was performed in discretion of the leading anaesthesiologist and in accordance with institutional guidelines. Coagulation factor administration was guided predominately by rotational thromboelastometry (ROTEM). Transoesophageal echocardiography was used during each surgical procedure to determine global cardiac function, regional wall motion abnormalities, valvular function, volume status, and deaeration before unclamping of the aorta.

Following surgery, the patients were transferred to the cardiovascular ICU, intubated, sedated, and mechanically ventilated. They were awakened and extubated when the extubation criteria were fulfilled which is an awake, cooperative patient, with completely reversed neuromuscular function, hemoglobin oxygen saturation >96%, with fraction of inspired O_2_ ≤ 0.4, end tidal CO_2_ 4–6 kPa, stable hemodynamics, and normal core temperature, with retrosternal drainage less than 100 mL/h. Postoperative pain was treated by continuous intravenous infusion of morphine and intravenous paracetamol.

Patients were transferred to the ward when they met the following criteria: hemoglobin oxygen saturation ≥94% at a fraction of inspired O_2_ of ≤0.4; hemodynamic stability without hemodynamically significant arrhythmias, without intravenous inotropic or vasopressor therapy, with diuresis >0.5 mL/kg/h, without delirium or epileptic activity, and without signs of infection.

Following patient discharge, follow-up was by telephone at 30 days after surgery, where the focus was on late postoperative morbidities and mortality.

### 2.3. Data Collection and Measurement Time Frame

We documented patient preoperative data (i.e., demographic characteristics, preoperative medical status, patient assessment according to European System for Cardiac Operative Risk Evaluation II (EuroSCORE II) for risk of death (http://www.euroscore.org/calc.html), and according to the American Society of Anaesthesiologists (ASA) classification) and intraoperative data (procedural times, blood loss, quantity of crystalloids, colloids, blood/blood components, and procoagulant factors administered, inotropic and/or vasoactive drugs, and insulin used). Furthermore, following postoperative data were also documented: duration of mechanical ventilation in the ICU, length of ICU and in-hospital stay, postoperative consumption of inotropic/vasoactive drugs and insulin, along with fluids, blood, and blood components, plus postoperative complications (e.g., bleeding, hemodynamic instability, impaired respiratory function, worsening of renal, liver, and brain functions, and infections), and 30-day mortality.

### 2.4. Outcome Measures

Primary outcome measures were for evolution of cytokine levels (TNF-*α*, IL-1*β*, IL-6, IL-8, and IL-10) and complement C5a, as well as expression of CD64 and CD163 markers on monocytes, granulocytes, and lymphocytes.

Secondary outcome measures were for changes in serum hs-CRP and procalcitonin levels, leukocyte count, albumin, fibrinogen, and hemodynamic measurements (i.e., cardiac index, systemic vascular resistance index, central venous oxygen saturation, and mean arterial pressure).

Other prespecified outcome measures included duration of postoperative mechanical ventilation, length of ICU stay, use of inotropic/vasoactive drugs, use of fluid/blood products and insulin, length of in-hospital stay, and 30-day mortality.

### 2.5. Blood Sampling

#### 2.5.1. Cytokine and Complement Analysis

For all the laboratory analyses, blood samples were collected without any additives. The serum was separated from the clotted blood by centrifugation (1,500 ×g for 10 min), and aliquots were stored at −20°C until analyzed. Serum TNF-*α*, IL-1*β*, IL-6, IL-8, and IL-10 was measured using chemiluminescent immunometric assays with an automated analyser (reagents and analyser: Immulite; Siemens Healthcare, Erlangen, Germany). The analytical sensitivity was 0.1 ng/L for IL-1*β* and 1 ng/L for the other measures. Serum aliquots used for the measurement of the complement component C5a were diluted 400-fold before analysis, with the measurements carried out using ELISA assays, with a detection limit of 31 ng/L (RayBiotech, Norcross, Georgia, USA).

### 2.6. CD64 and CD163 Expression Analysis

Whole blood from donors was collected in EDTA-containing vacutainer tubes (BD Vacutainer Systems, Franklin Lakes, New Jersey, USA) and stored at room temperature until used. Then, 100 *μ*L whole blood was stained with a mouse-anti-human CD64-FITC antibody (ThermoFisher Scientific, Waltham, Massachusetts, USA; ref: 11-0649-42), a mouse-anti-human CD163-PE antibody (ThermoFisher Scientific, Waltham, Massachusetts, USA; ref: 12-1639-42), and a mouse-anti-human CD14-PerCP-Cy5.5 antibody (BD Biosciences, San Jose, California, USA; ref: 550787) at room temperature for 20 min. After this incubation, 2 mL 1 × BD FACS Lysing Solution (BD Biosciences, San Jose, California, USA; ref: 349202) was added. After incubation at room temperature for 10 min, the samples were washed with 2 mL phosphate-buffered saline. Additionally, the samples were resuspended in 450 *μ*L phosphate-buffered saline. The CD64 and CD163 mean fluorescence intensity (MFI) on the monocytes, granulocytes, and lymphocytes was determined by FACS analysis (BD FACSCanto II; BD Biosciences, San Jose, California, USA).

### 2.7. Statistical Analysis

Demographic and clinical baseline data were summarized according to mean and standard deviation, or median and range, as expressed through minimum and maximum values, for metric variables, or to absolute frequencies for categorical variables.

As the Kolmogorov–Smirnov tests for normal distributions rejected the null hypothesis that majority of variables were normally distributed, the nonparametric Kruskal–Wallis tests were applied for independent samples along with median tests for independent samples [24]. Both these tests indicated that there were significant overall differences between groups for several of the variables (with *p* values given; Kruskal–Wallis tests). To analyse differences in greater detail, Mann–Whitney tests were applied for testing each pair of patient groups for different distributions. Variables with repeated measurements were analyzed using the Friedman test for related samples. Differences between individual pairs of measurements were further analyzed using the Wilcoxon signed rank test. The Spearman correlation test was used to test correlation between variables. The analyses were performed using SPSS v.25.0 software package (SPSS Inc., Chicago, IL, USA). Prior to determining significance, the Bonferroni correction for multiple comparisons was applied to the initially stated significance threshold of *p* value <0.05.

## 3. Results

**Figure 1:**
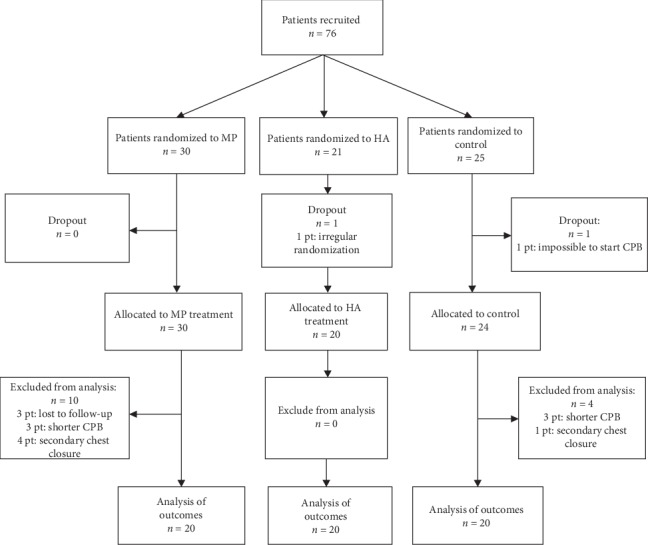
Flowchart of patients in the randomized trial.

In total, 76 patients were enrolled, with 16 patients dropping out of the study after randomization (6 patients had shorter CPB than planned, 5 patients had surgical complications with rethoracotomy and secondary chest closure, 1 patient had irregular randomization, 1 had heavy calcified big blood vessels and cannulation for CPB was impossible, and 3 patients were lost to follow-up) (Figure 1).

Sixty patients (68% male, 32% female) were included in final analysis, with 20 patients in each group. Baseline and perioperative characteristics are detailed in Table 1.

There were no significant differences among groups for patient age, gender, EuroSCORE II, ASA classification, LVEF%, type of surgery, duration of surgery, duration of CPB, and aortic cross-clamping. There were also no significant differences between groups for two different types of oxygenators used with CPB.

### 3.1. Primary Outcome Measures

**Figure 2:**
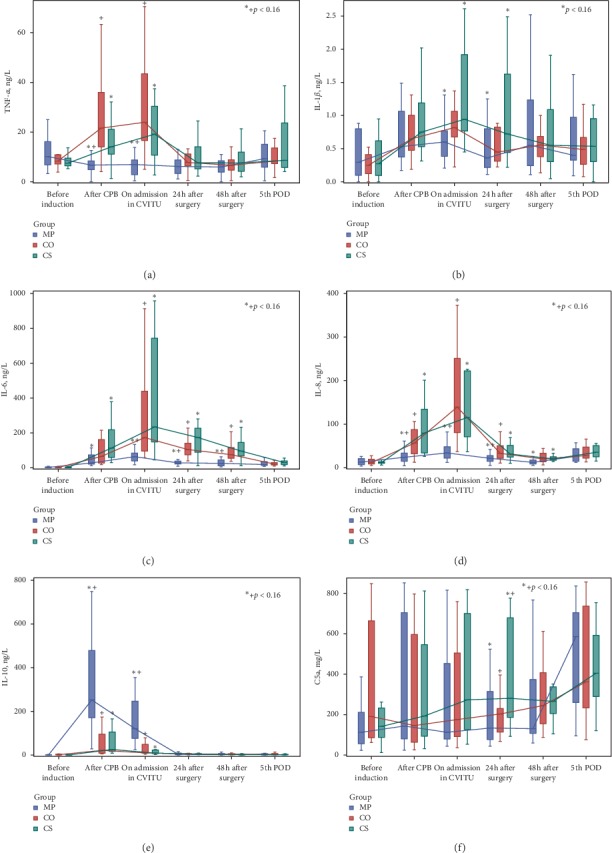
Cytokines and C5a complement.

**Figure 3:**
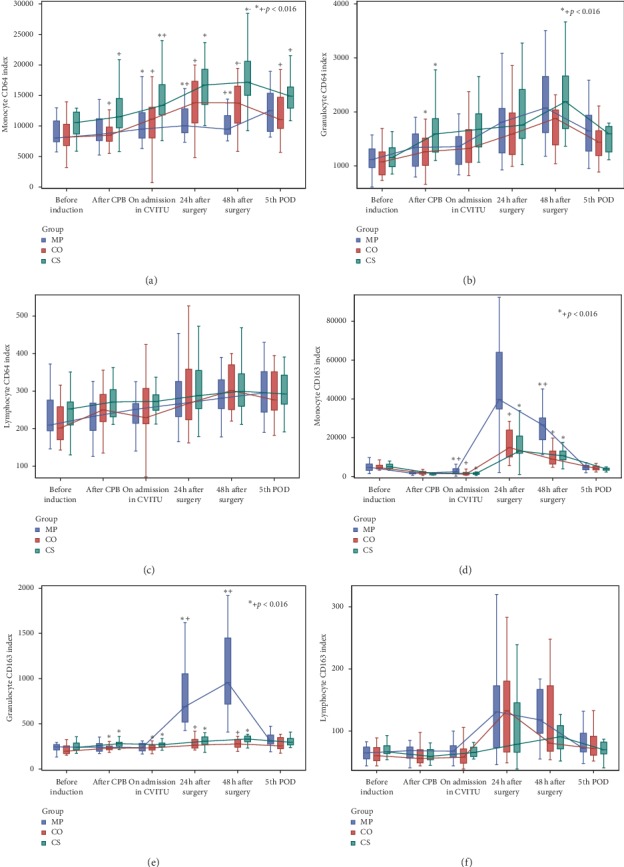
CD64 and CD163 expression on monocytes, granulocytes, and lymphocytes.

Levels of pro- and anti-inflammatory cytokines, C5a complement, and CD64 and CD163 expression on immune cells are presented in Figures 2 and 3 ([Supplementary-material supplementary-material-1] in Supplementary Material). The Methylprednisolone group, compared to the Cytosorb and Control groups, had significantly lower levels of TNF-*α* (until the end of surgery, *p* < 0.001), IL-6 (until 48 h after surgery, *p* < 0.001), and IL-8 (until 24 h after surgery, *p* < 0.016). CD64 expression on monocytes was the highest in the Cytosorb group and lasted until the 5^th^ POD (*p* < 0.016). IL-10 concentration (until the end of surgery) and CD163 expression on monocytes (until 48 h after surgery) were the highest in the Methylprednisolone group (*p* < 0.016, for all measurements between three groups).

Statistical significance between the Cytosorb and Control groups was found only for C5a (24 h after surgery, *p*=0.012), CD64 on granulocytes (after CPB, *p*=0.009), and CD64 on monocytes (after CPB, on ICU admission, 48 h after surgery, and 5^th^ POD: *p*=0.003, *p*=0.004, *p*=0.009, and *p*=0.007, respectively)—all higher in the Cytosorb group—as well as for anti-inflammatory CD163 on granulocytes (after CPB and on ICU admission—*p*=0.001 and *p*=0.006, respectively)—higher in the Cytosorb group.

Statistical significance in repeated measurements, i.e., differences between “before induction of anaesthesia” measurement and other individual measurements, was reached for all inflammatory mediators in the three study groups in some of the measurements, which could be seen in [Supplementary-material supplementary-material-1] in Supplementary Material.

### 3.2. Secondary Outcome Measures

**Figure 4:**
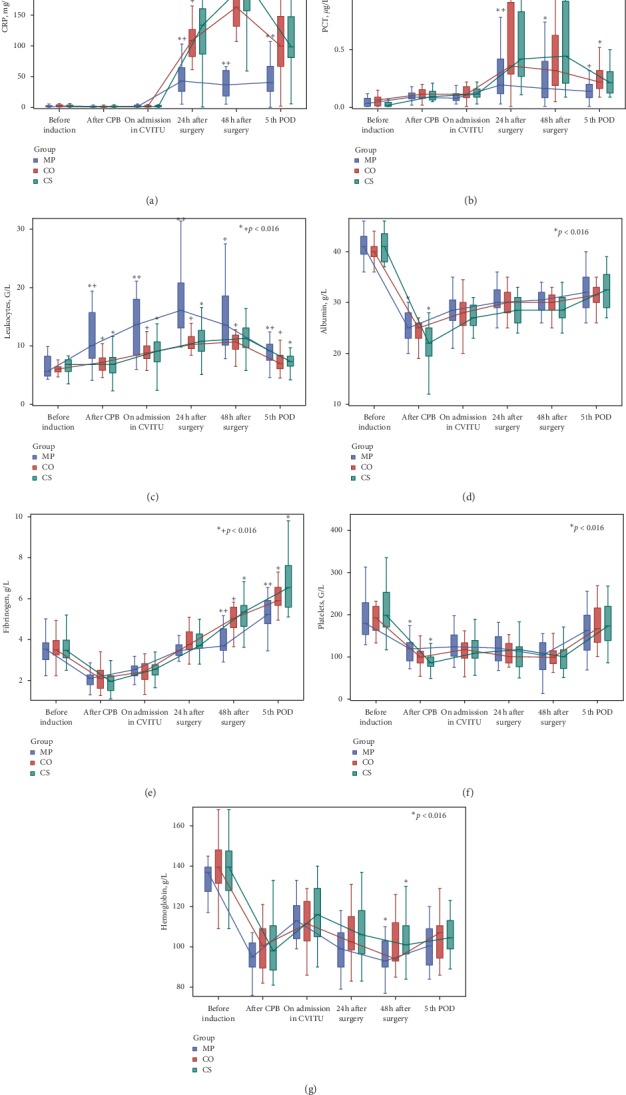
Laboratory secondary outcome measures.

Secondary outcome laboratory measures are presented in Figure 4 ([Supplementary-material supplementary-material-1] in Supplementary Material).

The Methylprednisolone group showed the greatest increases in leucocyte count and lowest levels of hs-CRP and PCT.

Albumin and fibrinogen showed few differences between treatment groups, with albumin generally slightly lower in the Cytosorb group (*p*=0.005 for MP *versus* CS after CPB). Overall, their levels decreased after CPB and then slowly increased thereafter. Platelet counts were at their lowest after CPB (*p*=0.003 for MP *versus* CS), without any other significant differences between treatment groups.

**Figure 5:**
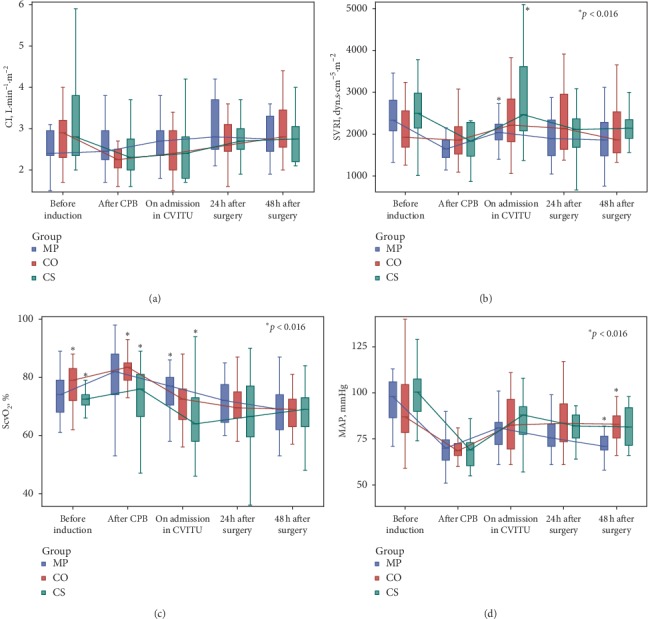
Hemodynamic parameters.

All patients remained hemodynamically stable (Figure 5) ([Supplementary-material supplementary-material-1] in Supplementary Material). The Methylprednisolone group generally showed the lowest systemic vascular resistance index (from after CPB on) and mean arterial pressure (from ICU admission on), with the highest cardiac index (from after CPB till 48 h after surgery).

Additional statistical analysis showed that of all the inflammatory mediators and biochemical parameters, in general, with duration of surgery, correlated only IL-6 (positive correlation for *p*=0.000; after Bonferroni correction for multiple comparisons significant *p* < 0.0001) ([Supplementary-material supplementary-material-1] in Supplementary Material 2).

We additionally analyzed the possible association between inflammatory markers and biochemical parameters (primary and secondary outcome measures) with different types of surgical procedures (listed in Table 1). To compare differences in parameters between different types of surgery, we applied the nonparametric Kruskal–Wallis test for comparison of independent samples. The *p* value (*p* < 0.0018 after Bonferroni correction for multiple comparisons) indicated no significant difference between none of the inflammatory mediators or biochemical parameters and different types of surgery (Tables [Supplementary-material supplementary-material-1] and [Supplementary-material supplementary-material-1] in Supplementary Material 2).

### 3.3. Other Prespecified Outcome Measures

No significant differences were seen between treatment groups for duration of postoperative mechanical ventilation, length of ICU and in-hospital stay, or 30-day mortality. Indeed, none of the patients died during the first month after surgery although one patient from the Methylprednisolone group died after the first month while still in hospital. This patient contracted a hospital-acquired infection of surgical wound (sternum), with consequent sepsis and multiorgan failure (Table 1).

Data of postoperative complications are presented in Table 1; there were no significant differences between groups.

**Figure 6:**
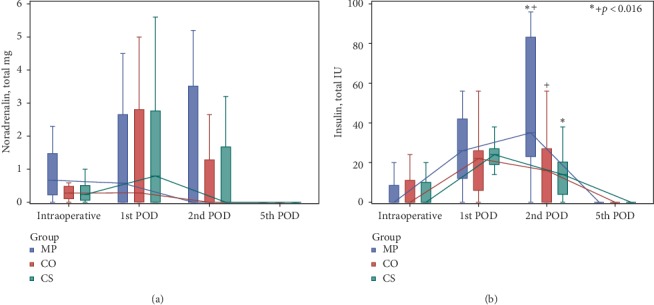
Consumption of noradrenalin (a) and insulin (b).

There was not any statistical significance for vasoactive/inotropic drug consumption between groups in any measurement time (Figure 6) ([Supplementary-material supplementary-material-1] in Supplementary Material). On the contrary, for insulin use, significant differences were seen 48 h after surgery for MP *versus* CS (*p* < 0.001) and MP *versus* CO (*p*=0.003) groups. Thus, the use of insulin was the highest in the Methylprednisolone group, as expected, while there were no significant differences between the Cytosorb and Control groups.

## 4. Discussion

In this clinical trial, we have confirmed that intraoperative use of median dose of methylprednisolone during CPB more effectively ameliorates systemic inflammatory responses after adult cardiac surgery. This is seen by reductions in proinflammatory and increases in anti-inflammatory mediators, when compared to both use of CytoSorb® cartridges for hemadsorption and usual treatment (Control). However, methylprednisolone did not provide a better short-term clinical outcome. Hemadsorption itself, compared with usual care, caused higher prolonged expression of CD64 on monocytes and higher expression of CD163 on granulocytes that only lasted until the end of surgery. Use of CytoSorb® seems to be safe and well tolerated. We have not observed association between its application and significant thrombocytopenia or more significant decrease in albumin concentration.

In contrast to our trial, data from a retrospective clinical study by Born et al. [21] that included 40 patients who underwent surgery of ascending aorta and aortic arch with selective brain perfusion showed positive effects of hemadsorption on inflammatory mediators after CPB. The reason for this might be the type of surgery with longer CPB time (224/213 min in their trial *versus* median times of 150/146/127 min in our three study groups).

Recently published data of Garau et al. [22] also showed minor and short lasting, but a significant reduction in proinflammatory cytokine levels of IL-8 and TNF*α* in cardiac surgery patients treated with hemadsorption. This cannot be explained by their CPB times, which are shorter than ours (133/128 min); additionally, flow through the CytoSorb® cartridge with 300 ml/min was less than that in our trial (400 ml/min); however, their patients had higher EuroScore-6, in comparison with ours (1.98/2.26/2.78) and slightly higher inflammatory response in relation to IL-6 than our patients (peak postoperative levels 263 *versus* 235 ng·L^−1^), which could be a reason for difference in this outcome.

The results of our trial are supported by two prospective randomized clinical trials, by Bernardi et al. [23] (on 37 patients, 18 with hemadsorption) and by Poli et al. [25] (on 30 patients, 15 with hemadsorption). Both showed no effects of hemadsorption with Cytosorb® during CPB, neither on ‘biochemical alleviation' of post-CPB SIRS, nor on patient clinical outcomes. The only effect Bernardi et al. saw was on IL-10, which showed prolonged elevated levels in patients treated with hemadsorption during CPB; however, this was not confirmed in our study.

Other available data in literature regarding use of hemadsorption in cardiac surgery are generally only case series or case reports. These appear to show the postoperative effects of hemadsorption use during CPB as decreased proinflammatory mediators [26–28].

For the high-affinity FcyRI receptor, CD64 is well known that it has potential utility as a marker for diagnostic assessment of SIRS and sepsis [29]. Thornton et al. [30] demonstrated that the hemoglobin scavenger receptor CD163 facilitates regulation and resolution of inflammation and removal of free hemoglobin and is highly expressed in myeloid cells from patients with inflammatory disorders, such as systemic juvenile idiopathic arthritis (SJIA) and macrophage activation syndrome (MAS). Recently, Comi at al. [31] demonstrated that coexpression of CD163 and CD141 identifies human-circulating IL-10-producing dendritic cells termed as DC-10 because of their ability to spontaneously secrete IL-10. CD163 has also been shown to be elevated in SIRS and sepsis in adults. Regulation of CD163 mRNA expression is a key functional property of polarized monocytes and macrophages.

Kolackova et al. [32] conducted a study with 40 “on-pump” and “off-pump” patients undergoing coronary artery bypass graft, and they concluded that there were substantial changes in expression of CD64 and CD163 in both these cardiac surgical patient groups during operation and in the postoperative period.

We have demonstrated that glucocorticoids decrease CD64 and increase CD163 expression after CPB, especially on monocytes. We have also detected prolonged higher expression of CD64 on monocytes after hemadsorption with CytoSorb® that lasted until the 5^th^ postoperative day. CD64 on monocytes is a biomarker reflecting type-I interferon (IFN) levels, which could potentially ameliorate autoimmune reaction (i.e., Dressler syndrome) after cardiac surgery [33–35].

There are conflicting data on the effects of Cytosorb® on the cytokine levels in septic shock and multiorgan failure. Although Cytosorb treatment has been shown to improve hemodynamics in these patients and most small nonrandomized studies also describe cytokine reductions (i.e., IL-6), this was not confirmed in a recently published randomized study by Schadler et al. [36]. Namely, in this multicenter randomized study, the authors reported that the treatment with Cytosorb in a population of severely ill patients with mainly septic shock, acute respiratory distress syndrome, and multiorgan failure led to hemodynamic improvement and substantial removal of IL-6 through the filter. However, this did not result in decreased IL-6 plasma levels compared with the control group. There were also no significant differences in secondary outcomes, i.e., in multiple organ dysfunction score and duration of mechanical ventilation and oxygenation.

As far as prophylactic use of glucocorticoids during CPB is considered, data in literature are numerous. Most studies, however, show no positive effects of glucocorticoid use on patient clinical outcome [37–39]. In the latest meta-analysis on 16, 013 patients, Dvirnik et al. [38] concluded that steroid administration at the time of cardiac surgery had an unclear impact on mortality and increased the risk of myocardial injury, and the impact on atrial fibrillation should be viewed with caution given that large trials showed no effect.

To the best of our knowledge, this is the first randomized clinical trial in literature that compares effects of hemadsorption *versus* methylprednisolone and usual care during CPB, in terms of levels of proinflammatory and anti-inflammatory mediators after open complex cardiac surgery and clinical outcome. Contrary to our expectations, we were able to demonstrate only subtle effects of hemadsorption on the modulation of SIRS after CPB. Nevertheless, use of CytoSorb® had a beneficial effect intraoperatively, as patients from the Cytosorb group with hemadsorption had the lowest need for norepinephrine, while the patients in the Methylprednisolone group had the highest; however, this was seen only during surgery and did not reach statistical significance. It correlates with systemic vascular resistance, which was the highest in the Cytosorb group and the lowest in the Methylprednisolone group, which consequently showed the lowest mean arterial pressure and highest cardiac index. The present study showed no beneficial effects of any of these interventions on patient clinical outcome.

Additionally, speaking in general for all study patients and regarding all inflammatory mediators and biochemical parameters, we found positive correlation only between IL-6 concentrations after CPB with duration of surgery. There are data in the literature regarding this, namely, some authors [40] found a positive correlation between the magnitude of the IL-6 response to CPB, but with duration of CPB (and not duration of aortic cross-clamp), while others reported that CPB duration correlated with IL-8 concentration [41] (we did not find this association). Others [23] did not find any correlation between cytokine peaks and treatment time.

As far as immune response and different types of surgical procedures in heart surgery are concerned, patients undergoing valve surgery appear to have similar immunologic response profiles to CABG patients [41]. Some authors report that, in general, indices of inflammation appear to correlate with overall severity of illness rather than a specific surgical procedure, which supports the findings from our trial. Namely, we did not find any association between inflammatory mediators and biochemical parameters and different types of surgical procedures. However, the statistical analysis we performed is somehow unreliable since we had some groups of only 1-2 patients, which is unevenly distributed and far too small for serious statistics. Nonetheless, this could be an idea for a next research that will examine exactly this phenomenon.

There are some major limitations to the present study, the biggest being it is a single-centre trial. Sample size is small and although sufficient for assessing primary and secondary outcomes, it does not allow definitive conclusions on the effects of study treatments on postoperative complications and patient clinical outcome. Moreover, effects of hemadsorption might have been more pronounced if duration of CPB was longer, or if only high-risk patients had been included (i.e., aortic arch surgery with hypothermic arrest and selective perfusion of brain, endocarditis surgery, and higher EuroSCORE II).

Future prospective randomized studies are needed to address the potential limitations discussed above. They should preferably evaluate the effectiveness of intraoperative hemadsorption in patients with a high inflammatory state, like those with infective endocarditis, emergency surgery, or patients for implantation of mechanical circulatory support and heart transplant.

## 5. Conclusions

Intraoperative glucocorticoids appear to be superior compared to hemadsorption or usual care for biochemical alleviation of systemic inflammation after CPB. However, the use of intraoperative glucocorticoids did not result in a better short-term outcome. Hemadsorption itself, compared with usual treatment, caused higher prolonged expression of CD64 on monocytes and higher expression of the anti-inflammatory marker CD163 on granulocytes that lasted only until the end of surgery, both which need further evaluation.

## Figures and Tables

**Table 1:** Patient and surgery characteristics at baseline.

Characteristic	Methylprednisolone (*n* = 20)	Cytosorb (*n* = 20)	Control (*n* = 20)	*p* value^*∗*^
Preoperative				
Age, years (median) [range]	66.5 (18.25) [43–80]	70.5 (26.5) [34–80]	71 (17.75) [31–85]	0.525/0.171/0.372
Male/female	13/7	14/6	14/6	0.736/0.736/1.000
Body surface area, m^2^ (median) [range]^ASA^	1.811 (0.206) [1.548–2.133]	1.962 (0.28725) [1.716–2.286]	1.97 (0.245) [1.652–2.257]	0.038/0.068/0.705
EuroSCORE II (median) [range]	1.985 (0.9925) [0.96–12.98]	2.26 (2.825) [0.96–10.43]	2.78 (4.645) [0.96–10.74]	0.579/0.203/0.645
LVEF, % (median) [range]	61.5 (14) [29–87]	60.5 (11.25) [30–80]	65 (20) [39–77]	0.249/0.683/0.414
Intraoperative				
One valve surgery + CABG, M/F	5/3	0/3	6/3	0.077/0.749/0.038
More than one valve surgery + CABG, M/F	2/0	0/0	1/0	0.147/0.548/0.311
More than one valve surgery, M/F	1/3	4/2	1/2	0.465/0.677/0.256
Surgery of ascending aorta, M/F	1/1	5/0	4/0	0.212/0.376/0.705
Valve surgery + CABG + surgery of ascending aorta, M/F	1/0	1/0	0/0	1.000/0.311/0.311
Valve surgery + surgery of ascending aorta, M/F	2/0	3/1	1/1	0.376/1.000/0.376
Valve surgery + other procedures (ASD and RFA), M/F	1/0	0/0	1/0	0.311/1.000/0.311
Valve surgery + surgery of ascending aorta + other procedure (ASD and RFA), M/F	0/0	1/0	0/0	0.311/1.000/0.311
Anaesthesia time, min (median) [range]	358 (91.25) [249–563]	352.5 (124.25) [237–499]	335 (81.5) [258–528]	0.989/0.561/0.425
Surgery time, min (median) [range]	245.5 (74.5) [150–445]	252.5 (101.75) [153–388]	237 (59) [161–393]	0.766/0.457/0.317
Cardiopulmonary bypass time, min (median) [range]	150.5 (41.5) [90–240]	146 (68.5) [91–281]	127 (56.75) [90–238]	0.725/0.239/0.473
Aorta cross-clamping time, min (median) [range]	104 (43.5) [66–206]	105 (45) [56–244]	102 (43.5) [63–184]	0.882/0.482/0.725
Erythrocytes, mL (median) [range]	0 (308.75) [0–585]	0 (567.5) [0–1525]	0 (575) [0–1760]	0.495/0.176/0.748
Fresh frozen platelets, mL (median) [range]	0 (934.5) [0–1322]	527.5 (768) [0–1031]	503 (738.75) [0–1282]	0.346/0.616/0.415
Thrombocytes, mL (median) [range]	0 (0) [0–350]	0 (0) [0–300]	0 (0) [0–0]	0.971/0.317/0.317
Cell saver, mM (median) [range]	580 (520) [0–2927]	467 (358.25) [0–1147]	511.5 (305.25) [0–1000]	0.099/0.285/0.379
Fibrinogen, g (median) [range]	0 (2) [0–4]	1 (2) [0–3]	0 (2) [0–3]	0.834/0.482/0.558
Prothrombin complex concentrate, IE (median) [range]	0 (0) [0–3000]	0 (0) [0–500]	0 (0) [0–0]	0.264/0.076/0.317
Recombinant activated factor VII, mg (median) [range]	0 (0) [0–7]	0 (0) [0–0]	0 (0) [0–0]	0.317/0.317/1.000
Crystalloids, mL (median) [range]	1500 (500) [1000–2000]	1500 (1000) [1000–2500]	1000 (500) [1000–2000]	0.098/0.696/0.069
Colloids, mL (median) [range]	0 (0) [0–1000]	0 (0) [0–500]	0 (500) [0–500]	0.150/0.386/0.021
Postoperative				
Postoperative mechanical ventilation, h (median) [range]	9.5 (11) [3–1257]	14.5 (11.75) [4–26]	8.5 (12.25) [5–29]	0.192/0.514/0.568
Length of stay (ITU), h (median) [range]	90.5 (132) [58–1932]	121 (77) [47–304]	117 (89.5) [64–407]	0.379/0.465/0.756
Length of stay (hospital), days (median) [range]	10 (9) [6–82]	13 (8.25) [6–30]	11 (11) [6–26]	0.497/0.644/0.625
New-onset atrial fibrillation	4	5	3	0.705/0.677/0.429
Worsening of renal function	0	0	1	1.000/0.311/0.311
Postoperative myocardial infarction	0	0	1	1.000/0.311/0.311
Postoperative delirium	0	2	0	0.147/1.000/0.147
Dressler syndrome	3	0	2	0.072/0.633/0.147
Tamponade	3	1	1	0.292/0.292/1.000
Thrombocytopenia	1	1	0	1.000/0.311/0.311
Infection	2	4	4	0.376/0.376/1.000
Without postoperative complications	7	6	4	0.736/0.288/0.465
30-day mortality	0	0	0	1.000/1.000/1.000
30-day readmission	2	3	3	0.633/0.633/1.000
1-year mortality	1	0	0	0.311/0.311/1.000

Data are shown as (median) and [interquartile range]. MP, methylprednisolone; CS, cytosorb; CO, control. ^*∗*^*p* value for comparisons of groups: (MP *versus* CS)/(MP *versus* CO)/(CO *versus* CS).

## Data Availability

The table data used to support the findings of this study are included within Supplementary Materials.

## Supplementary Materials

Supplementary Materials Table 2: levels of cytokines, C5a complement, and CD163. Table 3: laboratory secondary outcome measures. Table 4: hemodynamic parameters. Table 5: consumption of vasoactive/inotropic drugs and insulin; CONSORT 2010 checklist of information to include when reporting a randomised trial. Table 6: correlations between inflammatory and biochemical parameters with four procedural times; Spearman's correlation coefficient (*p* value = 0.0001 after Bonferroni correction for multiple comparisons). Table 7: Kruskal–Wallis test for testing the association between inflammatory mediators and biochemical parameters with eight different types of surgical procedures (*p* value = 0.0018 after Bonferroni correction for multiple comparisons). Table 8: range values in groups of different surgical procedures.
