# Supplementary material for: Extracorporeal Hemadsorption versus Glucocorticoids during Cardiopulmonary Bypass: A Prospective, Randomized, Controlled Trial
Source: Cardiovasc Ther. 2020 Mar 27;2020:7834173. doi: 10.1155/2020/7834173 (PMC7149340; doi:10.1155/2020/7834173)
Supplement: Supplementary Materials — Table 2: levels of cytokines, C5a complement, and CD163. Table 3: laboratory secondary outcome measures. Table 4: hemodynamic parameters. Table 5: consumption of vasoactive/inotropic drugs and insulin; CONSORT 2010 checklist of information to include when reporting a randomised trial. Table 6: correlations between inflammatory and biochemical parameters with four procedural times; Spearman's correlation coefficient (p value = 0.0001 after Bonferroni correction for multiple comparisons). Table 7: Kruskal–Wallis test for testing the association between inflammatory mediators and biochemical parameters with eight different types of surgical procedures (p value = 0.0018 after Bonferroni correction for multiple comparisons). Table 8: range values in groups of different surgical procedures. [file 7834173.f1.pdf]

Supplementary Material

Table 2. Levels of cytokines, C5a complement and CD64 and CD163.

Measure	Group	Before induction of anesthesia	After CPB	After surgery (i.e. on admission in ICU)	24 h after surgery	48 h after surgery	Postoperative day 5
TNF-α, ng/L	MP	10.15 (9.7175) [3.28-58]	6.72 (3.8025) [0-29.9]*	6.965 (6.1225) [0.419-31]*	6.19 (6.02) [1.14-45.5]	5.935 (4.8775) [0-53.2]	9.32 (10.645) [0.421-70.1]
(median) [range]	CS	7.51 (3.3) [5.27-64.8]	14.05 (10.71) [1.31-851]*	19.15 (20) [2.78-947]*	7.61 (9.9275) [2.26-41.7]	7.6 (8.2225) [2.03-47.8]	8.64 (20.8075) [4.06-97.9]
	CO	8.31 (3.8025) [3.89-45.2]	21.6 (22.75) [4.12-92.8]*	23.95 (28.7) [5-586]*	7.77 (4.8375) [0.564-73.6]	6.65 (4.6175) [0.481-21.7]	8.325 (7.3275) [1.68-44.7]
**p value**		0.223/0.646/0.304	**0.000**/**0.000**/0.099	**0.000**/**0.000**/0.176	0.160/0.057/0.655	0.441/0.465/0.725	0.655/0.989/0.695
IL-1β, ng/L	MP	0.295 (0.72375) [0-2.21]	0.5315 (0.76625) [0.17-2.32]	0.6015 (0.41825) [0.206-8.3]	0.3595 (0.6255) [0.11-74.1]	0.553 (1.0165) [0.109-3.24]	0.4 (0.706) [0.091-3.35]
(median) [range]	CS	0.2715 (0.5465) [0-4.04]	0.7495 (0.69725) [0.314-3.27]*	0.9435 (1.1935) [0.45-8.16]*	0.722 (1.277) [0.219-6.66]*	0.5505 (0.856) [0.048-6.56]	0.5365 (0.6615) [0-2.06]
	CO	0.2495 (0.303) [0-1.6]	0.6485 (0.543) [0.189-2.37]*	0.8185 (0.45775) [0.223-2.57]*	0.4475 (0.52) [0.225-9.24]*	0.555 (0.34975) [0.137-48]	0.4875 (0.4175) [0.077-8.41]
**p value**		0.694/0.533/0.616	0.168/0.473/0.534	**0.001**/0.047/0.11	**0.015**/0.344/0.117	0.903/0.685/0.766	0.978/0.607/0.588
IL-6, ng/L	MP	2.59 (4.69) [0-9.17]	28.15 (56) [12.2-174]*	64.05 (50.2) [17.2-134]*	28.55 (18.975) [9.34-68.1]*	25.8 (35.95) [5.6-125]*	18.95 (23.125) [8.31-295]*
(median) [range]	CS	1.925 (4.49675) [0-7.25]	113.5 (169.575) [30.4-1122]*	235 (649.5) [45.1-6908]*	174 (141.175) [12.1-281]*	96.2 (80.425) [11.7-233]*	30.45 (21.525) [10.8-55.8]*
	CO	2.305 (4.06875) [0.164-17.7]	65.35 (158.775) [18.4-1791]*	173.5 (373) [57.7-2359]*	105 (71.45) [39.8-501]*	75.6 (63.2) [37.8-254]*	21.9 (13.3) [9.95-43.7]*
**p value**		0.704/0.935/0.797	**0.000**/0.055/0.074	**0.000**/**0.000**/0.245	**0.000**/**0.000**/0.099	**0.000**/**0.000**/0.425	0.402/0.935/0.105
IL-8, ng/L	MP	13 (14.255) [3.95-143]	24.2 (21.125) [4.66-263]	34.05 (27.5) [12.3-82.2]	21.7 (13.45) [5.3-1357]*	12.45 (9.3725) [5.38-391]	26.35 (32.9) [12.5-104]
(median) [range]	CS	12.3 (3.7) [6.33-23.3]	79.85 (113.3) [26.1-780]*	115.5 (153.15) [36.9-4522]*	31.65 (30.825) [10.5-270]*	20.4 (8.275) [14.1-42.8]*	35.5 (26.475) [15.3-430]*
	CO	11.9 (11.345) [5.38-138]	56.6 (65.35) [12.6-390]*	139.5 (174.825) [37.2-1214]*	33.25 (30.275) [10.8-304]*	20.2 (20.175) [6.72-170]	28.15 (27.075) [13.4-177]
**p value**		0.675/0.968/0.957	**0.000**/**0.002**/0.433	**0.000**/**0.000**/0.829	**0.009**/**0.016**/0.957	**0.002**/0.032/0.675	0.107/0.441/0.330
IL-10, ng/L	MP	1.13 (1.28225) [0-6.22]	252.5 (330) [28.8-749]*	121 (175.025) [25-355]*	3.36 (7.865) [0.225-56.5]*	2.415 (5.3975) [0.454-37.4]*	3.06 (5.5475) [0.34-55.2]*
(median) [range]	CS	0.9125 (1.32975) [0-45.1]	25.85 (114.65) [9.01-464]*	9.175 (16.76) [2.73-1621]*	4.245 (3.555) [1.51-23]*	2.815 (2.7925) [0.481-14.5]*	1.98 (2.4825) [0.719-10]
	CO	2.04 (2.89) [0.4-19.3]	21.85 (87.89) [5.74-362]*	10.1 (55.705) [4.07-578]*	5.83 (2.705) [0.887-45.8]*	3.9 (5.295) [1.14-33.6]	3.11 (6.5575) [0.811-17.7]
**p value**		0.473/0.032/**0.010**	**0.000**/**0.000**/0.465	**0.000**/**0.000**/0.579	0.839/0.245/0.117	0.598/0.208/0.062	0.402/0.507/0.094
C5a, mg/L	MP	112.75 (158.95) [23.4-631.2]	143.85 (625.425) [24.3-852]	112.85 (461.3) [43.9-816]	133.95 (262.025) [43.9-777.2]	130.5 (341.925) [60-804]	585.75 (474.725) [95.2-836]*
(median) [range]	CS	141.75 (155.875) [12.39-864]	193.6 (528.55) [32-812]	273.45 (575.975) [54.2-818]	280.85 (507.375) [93-775.9]*	265.75 (146.575) [105.4-808]	404.95 (325.275) [120.4-754.3]*
	CO	190.85 (590.6) [62.4-848]	146.05 (551.325) [25.5-796.4]	175.85 (474.475) [36.5-759.2]	203.35 (119.775) [67.9-794.3]	244.95 (281.05) [88.1-836]	362.55 (530.8) [76.2-856]
**p value**		0.425/0.042/0.279	0.935/0.516/0.482	0.070/0.646/0.160	**0.009**/0.372/**0.012**	0.019/0.055/0.449	0.330/0.665/1.000
CD64mon MFI	MP	8042 (3619.5) [5747-20347]	8613(3891) [5250-67784]	9504 (4401.25) [6307-18097]+	10049 (4197.5) [7350-16139]	9464.5 (3171.015) [7544-14411]	12529.5 (6468.75) [8172-18955]*
(median) [range]	CS	10571 (3653) [5873-19633]	11498 (5171.5) [5808-20863]	13362.5 (5109.5) [7587-23991]*	16716.5 (5922.5) [10060-23678]*	17176 (5649.25) [9224-28441]*	14881.5 (3591.5) [10845-21511]*
	CO	8164 (3943.03) [3203-13962]	8476 (2404.5825) [3578-13873]	11116 (5508.25) [707-18080]*	13803 (6995.25) [4818-19983]*	13784.525 (6162.5) [5837-19451]*	11045 (5129) [5676.38-19269]*
**p value**		0.117/0.570/0.025	0.037/0.646/**0.003**	**0.001**/0.626/**0.004**	**0.000**/**0.010**/0.042	**0.000**/**0.001**/**0.009**	0.045/0.626/**0.007**
CD64gran MFI	MP	1118.43 (363.25) [609-3515]	1342.5 (621.5) [793-3332]	1356 (526.8625) [835-3168]*	1811.5 (847.4) [926-3084]*	2074.5 (1068.705) [1180-4417]*	1647.5 (670.75) [951-5640]*
(median) [range]	CS	1154.5 (375.75) [851-2624]	1591.5 (642.75) [1100-2780]*	1676.5 (638.5) [1067-3290]*	1756 (1053.25) [1022-6360]*	2190.5 (1024.75) [1366-6021]*	1595.5 (480) [1115-3096]*
	CO	1073.5 (445.25) [728-1693]	1263.045 (506.75) [660-1866]*	1322.5 (639.5) [821-2374]*	1590 (792) [991-2858]*	1879.5 (677.75) [1041-4958]*	1439.89 (489.5) [887.76-3011]*
**p value**		0.745/0.402/0.185	0.035/0.695/**0.009**	0.020/0.914/0.025	0.417/0.665/0.152	0.646/0.105/0.040	0.465/0.279/0.330
CD64lym MFI	MP	209.5 (84.25) [146-372]	232.5 (78.5) [126-416]	251.5 (56) [140-352]	265.89 (96.25) [165-477]	281 (81.7275) [178-390]*	295 (125.5) [190-531]*
(median) [range]	CS	252.5 (62.5) [130-351]	271 (73.25) [211-496]	272.5 (44) [212-381]	288 (106.75) [179-473]*	299.5 (92.25) [211-533]*	292.5 (86) [191-904]*

	CO	201.5 (88.75) [143-316]	250 (77.75) [135-470]*	229 (95.5) [72-424]*	267.37 (138.5) [162-625]*	301.5 (128.25) [220-824]*	276.575 (107.5) [182-395]*
**p value**		0.372/0.194/0.047	0.021/0.337/0.180	0.066/0.957/0.117	0.409/0.787/0.665	0.490/0.441/0.745	0.685/0.626/0.409
CD163mon MFI	MP	4770 (3502.5) [1464-13293]	1648 (1162.25) [590-7021]*	2488.5 (2393.01) [178-7733]*	39687.48 (29775.75) [2110-92358]*	26582.5 (12360.5) [11278-70073]*	4692 (2398.5) [2032-12657]
(median) [range]	CS	5091.5 (2616.25) [3186-8042]	1636 (902) [749-10053]*	1650.5 (850.75) [571-3704]*	13199 (9429.5) [1099-50170]*	10734.5 (4666.25) [3949-29637]*	3946 (1145) [2327-6110]*
	CO	4588 (2017.59) [3266-8594]	1900 (1449.75) [522-5495]*	1240.5 (1438.5225) [627-11334]*	14920 (14151) [5565-52062]*	8968.12 (6955) [4861-26643]*	4254.78 (2046.5) [2371-6797]
**p value**		0.465/0.871/0.665	0.570/0.499/0.152	**0.010**/**0.015**/0.570	**0.000**/**0.000**/0.935	**0.000**/**0.000**/0.279	0.066/0.482/0.279
CD163gran MFI	MP	244.5 (63.5) [132-364]	240.39 (85.25) [171-353]	237.5 (91.25) [165-832]	690.5 (568) [426-1621]*	956 (737) [408-2928]*	304.5 (105.9875) [191-473]*
(median) [range]	CS	238.5 (78.5) [173-357]	280.5 (49) [216-428]	269 (43) [208-428]	306.5 (90) [184-898]*	332 (67.25) [202-1457]*	296.5 (85.75) [238-408]*
	CO	197.5 (96.5) [153-391]	229.5 (44.1075) [185-625]	234 (57) [170-359]	263 (100.5) [209-930]*	277.4 (92.25) [196-42722]*	255.5 (131.5) [175-385]*
**p value**		0.957/0.107/0.076	0.019/0.818/**0.001**	0.040/1.000/**0.006**	**0.000**/**0.000**/0.117	**0.000**/**0.000**/0.024	0.351/0.027/0.086
CD163lym MFI	MP	65 (22.5) [44-119]	68.565 (19.5) [41-551]	67.72 (20.25) [44-100]	131 (104.25) [46-376]*	118 (79.75) [55-1356]*	71.995 (31.5) [48-153]
(median) [range]	CS	66.5 (15.25) [41-133]	59.5 (16.75) [45-108]	65.5 (16) [55-82]	79 (88.75) [39-289]	91 (41.25) [52-168]*	70 (20) [41-437]
	CO	60.5 (21.5) [44-113]	56 (21.5) [44-134]	58 (25.0325) [39-142]	133 (117.62) [49-283]*	80.5 (107.6575) [54-651]*	72 (31.8225) [52-186]
**p value**		0.203/0.636/0.133	0.386/0.189/0.424	0.787/0.176/0.104	0.194/0.860/0.330	0.017/0.239/0.695	0.285/0.776/0.507

Data are shown as (median) and [interquartile range]

MP, Methylprednisolone; CS, Cytosorb; CO, Control; mon, monocytes; gran, granulocytes; lym, lymphocytes; MFI, mean fluorescence intensity from FACS analysis.

p values- are shown for comparisons of groups at different time points: (MP vs. CS)/(MP vs. CO)/(CO vs. CS); p = 0.000 defines p <0.001; significant p values are indicated in **bold text** (for $p<0.016$).

* $p < 0.01$ for statistical significance in repeated measurements (differences between 'before induction' measurement and other individual measurements).

Table 3. Laboratory secondary outcome measures.

Measure	Group	Before induction of anesthesia	After CPB	After surgery (i.e. on admission in ICU)	24 h after surgery	48 h after surgery	Postoperative day 5
C-reactive protein, mg/L, (median) [range]	MP	1.61 (2.36) [0.17-6.94]	1.09 (1.6) [0.17-5.44]*	1.43 (3.2375) [0.36-5.54]	42.85 (39.0) [5.44-124]*	36.7 (42.6) [5.44-127]*	40.6 (43.6) [0.165-161]*
	CS	1.715 (2.9825) [0.33-15.6]	1.24 (1.72) [0.2-9.34]*	1.76 (1.945) [0.27-12.5]	133.5 (80.5) [0.72-188]*	204 (85.5) [5.21-291]*	98.55 (71.2) [6.02-204]*
	CO	2.555 (3.3025) [0.38-66]	1.45 (1.80) [0.173-12.4]*	2.03 (2.5125) [0.57-13.2]	109 (45.9) [5.95-165]*	163 (58.75) [5.15-233]*	99.7 (84.05) [2-190]*
**p value**		0.482/0.25/0.705	0.735/0.871/0.989	0.465/0.579/0.946	**0.001**/**0.000**/0.152	**0.000**/**0.000**/0.058	**0.002**/**0.002**/0.935
Procalcitonin, µg/L (median) [range]	MP	0.035 (0.07) [0.009-0.12]	0.09 (0.055) [0.02-0.18]*	0.08 (0.065) [0.03-0.33]*	0.19 (0.37) [0.03-1.04]*	0.165 (0.323) [0.01-0.74]*	0.14 (0.123) [0.01-0.51]*
	CS	0.02 (0.0375) [0.009-0.07]	0.095 (0.077) [0.05-0.21]*	0.115 (0.075) [0.03-0.44]*	0.42 (0.61) [0.11-16.6]*	0.445 (0.73) [0.09-12.52]*	0.215 (0.19) [0.09-2.36]*
	CO	0.065 (0.0775) [0.009-0.15]	0.115 (0.0825) [0.02-0.3]*	0.115 (0.1025) [0.009-0.69]*	0.36 (0.76) [0.01-2.75]*	0.32 (0.4875) [0.05-1.82]*	0.22 (0.163) [0.09-0.52]*
**p value**		0.323/0.306/0.026	0.765/0.212/0.464	0.085/0.116/0.935	**0.011**/**0.007**/0.675	**0.002**/0.017/0.507	0.074/**0.012**/0.665
Leukocytes, G/L (median) [range]	MP	5.65 (3.45) [4.3-9.9]	10.1 (7.8) [4.1-19.4]*	13.65 (9.625) [6-21.1]*	16.1 (7.7) [9.8-31.4]*	13.6 (8.65) [7.8-27.5]*	9.15 (2.875) [4.6-15.1]*
	CS	6.8 (2.05) [3.5-8.3]	6.85 (2.675) [2.3-12.9]	9.1 (3.5) [1.9-16.2]*	10.8 (3.825) [5.1-20.1]*	11.3 (3.475) [5.8-18.5]*	7.35 (1.8) [3.5-12.7]
	CO	6.05 (0.999) [3.4-12.5]	7.2 (2.3) [4.6-15.3]	8.8 (2.525) [5.8-18.2]*	10.25 (2.08) [5.9-15.2]*	10.7 (2.5) [6.5-12.8]*	7 (2.4) [4.5-11]
**p value**		0.465/0.807/0.113	**0.004**/**0.005**/0.645	**0.007**/**0.010**/0.935	**0.000**/**0.000**/0.735	0.086/**0.015**/0.198	**0.014**/**0.013**/0.735
Albumin, g/L (median) [range]	MP	41 (3.75) [36-46]	25 (5) [20-36]*	28.5 (4.5) [21-35]*	30 (3.75) [25-36]*	30.5 (3.75) [23-34]*	32 (6) [26-40]*
	CS	41 (5.75) [37-46]	22 (5.75) [12-28]*	27 (4.5) [18-31]*	28.5 (5) [24-33]*	28.5 (4) [24-34]*	32.5 (6.75) [27-39]*
	CO	40 (2) [34-46]	25 (3) [19-32]*	28 (4.75) [20-37]*	30 (4) [25-35]*	30 (3.5) [25-33]*	31.5 (3.5) [26-38]*
**p value**		0.673/0.190/0.445	**0.005**/0.299/0.036	0.281/0.683/0.643	0.146/0.946/0.168	0.097/0.487/0.186	0.892/0.623/0.505
Fibrinogen, g/L (median) [range]	MP	3.53 (0.8825) [2.23-5.01]	2.1 (0.47) [1.09-2.86]*	2.53 (0.54) [1.41-3.18]*	3.46 (0.5875) [2.2-4.2]	3.685 (1.1025) [2.9-5.17]	5.235 (1.25) [3.45-8.1]*
	CS	3.475 (0.94) [2.5-5.2]	1.95 (0.7375) [1.1-2.98]*	2.545 (0.535) [1.5-3.39]*	3.7 (0.715) [2.8-5.55]*	5.35 (1.0775) [3.62-6.83]*	6.535 (2.078) [5.1-9.81]*
	CO	3.5 (0.7625) [2.23-4.94]	2.1 (0.9125) [1.26-3.4]*	2.44 (0.7925) [1.32-3.3]*	3.76 (0.9725) [2.8-5.09]	5.04 (1.05) [3.65-5.83]*	5.91 (0.927) [4.15-9.32]*
**p value**		0.914/0.695/0.957	0.448/0.653/0.223	0.645/0.675/0.490	0.030/0.030/0.968	**0.000**/**0.000**/0.417	**0.001**/**0.009**/0.223
Hemoglobin, g/L (median) [range]	MP	137 (13) [105-160]	95 (12) [76-138]*	113 (17.25) [99-133]*	99 (17) [79-118]*	93 (13) [77-110]*	100.5 (18.5) [84-120]*
	CS	139.5 (22.25) [109-168]	98 (23.5) [81-133]*	116 (24) [90-140]*	106 (22.75) [83-137]*	101 (15) [84-130]*	104.5 (14) [89-123]*
	CO	139.5 (19.25) [109-168]	100.5 (19.75) [82-121]*	111.5 (20.75) [86-129]*	102.5 (17.25) [83-131]*	94 (19.5) [85-126]*	107 (16.5) [86-129]*
**p value**		0.316/0.233/0.946	0.757/0.465/0.807	0.407/0.766/0.261	0.092/0.152/0.892	**0.016**/0.207/0.213	0.129/0.336/0.507
Platelets, g/L (median) [range]	MP	179.5 (75.75) [6-313]	119 (46) [72-174]*	124 (50) [75-198]*	121 (59) [68-182]*	104 (66) [13-155]*	163 (83) [69-256]*
	CS	198.5 (92.5) [117-335]	86 (24.5) [49-132]*	109 (42) [56-189]*	117.5 (49.5) [50-183]*	100.5 (47.25) [51-171]*	173.5 (85.5) [86-432]
	CO	192.5 (60) [133-232]	99.5 (30.5) [54-150]*	117.5 (36.75) [53-162]*	101 (52.25) [76-153]*	99 (31.75) [63-156]*	167.5 (84.25) [101-269]
**p value**		0.379/0.968/0.372	**0.003**/0.072/0.144	0.161/0.152/0.989	0.279/0.273/0.925	0.946/0.989/0.860	0.191/0.273/0.766

MP, Methylprednisolone; CS, Cytosorb; CO, Control

p values- are shown for comparisons of groups at different time points: (MP vs. CS)/(MP vs. CO)/(CO vs. CS); p = 0.000 defines $p < 0.001$; significant p values are indicated in **bold text** (for $p<0.016$).

* $p < 0.01$ for statistical significance in repeated measurements (differences between 'before induction' measurement and other individual measurements).

Table 4. Hemodynamic parameters.

Measure	Group	Before induction of anesthesia	After CPB	After surgery (i.e. on admission in ICU)	24 h after surgery	48 h after surgery
Cardiac index, L.min $^{-1}$.m^{-2} (median) [range]	MP	2.4 (0.65) [1.5-4.4]	2.45 (0.75) [1.7-3.8]	2.7 (0.65) [1.8-4.2]	2.8 (1.3) [2.1-5.8]	2.75 (0.925) [1.9-5.3]
	CS	2.8 (1.525) [2-6.5]	2.3 (0.825) [1.6-3.7]*	2.4 (1.05) [1.7-4.2]*	2.7 (0.55) [1.9-6.7]	2.75 (0.925) [2.1-5.8]
	CO	2.9 (0.95) [1.7-5.5]	2.25 (0.475) [1.6-3.4]	2.4 (1.075) [1.5-4.7]	2.6 (0.775) [1.6-4.3]	2.8 (0.95) [2-4.4]
**p value**		0.231/0.471/0.378	0.154/0.116/0.807	0.112/0.158/0.734	0.431/0.212/0.515	0.684/0.664/0.480
Systemic vascular resistance index, dyn.s.cm^{-5}.m^{-2} (median) [range]	MP	2333 (784.75) [1325-3460]	1643.5 (431) [1144-2558]*	2043.5 (382.75) [1062-3088]	1895.5 (911) [1049-2875]*	1860 (871.25) [757-3118]*
	CS	2496 (890.25) [1011-4568]	1832.5 (816.5) [868-4220]	2464 (1570.25) [1370-5093]	2108.5 (753.3) [667-3087]	2143.5 (457.5) [650-3407]
	CO	1924 (876) [1257-3235]	1855 (824.5) [1093-3078]	2216 (1043.5) [1059-4463]	2134 (1383.75) [1380-3913]	1864.5 (1008.25) [1325-3653]
**p value**		0.682/0.102/0.076	0.242/0.229/1.000	**0.015**/0.213/0.317	0.534/0.123/0.402	0.358/0.756/0.465
Central venous O_2 saturation, % (median) [range]	MP	74 (11) [61-98]	82 (14) [53-98]	77 (10.25) [58-86]	72 (13.5) [60-85]	69 (13) [53-87]
	CS	72.5 (3.75) [57-81]	76 (15.25) [47-89]	64 (17) [46-94]	66.5 (17.75) [36-90]	69 (10) [48-84]
	CO	79 (11) [62-88]	83.5 (6.5) [36-97]	72.5 (11.75) [56-94]	69.5 (9.5) [47-98]	69 (10.75) [57-90]
**p value**		0.290/0.242/**0.010**	0.058/0.811/**0.013**	**0.005**/0.176/0.081	0.239/0.655/0.440	0.957/0.903/0.989
Mean arterial pressure, mmHg (median) [range]	MP	98 (20.75) [71-113]	70 (12) [51-90]*	81 (13.5) [43-101]*	75.5 (12) [61-99]*	71 (7.75) [58-96]*
	CS	100.5 (18.25) [74-129]	69 (13.25) [55-100]*	88 (16) [57-108]*	82 (13.75) [64-110]*	81.5 (21.25) [39-98]*
	CO	87 (28.5) [59-140]	68.5 (7.25) [50-83]*	82.5 (28.5) [61-111]	83.5 (21.25) [61-117]	83 (12.5) [66-98]
**p value**		0.560/0.330/0.126	0.818/0.935/0.797	0.081/0.416/0.655	0.250/0.091/0.401	0.025/**0.008**/0.957

MP, Methylprednisolone; CS, Cytosorb; CO, Control

p values- are shown for comparisons of groups at different time points: (MP vs. CS)/(MP vs. CO)/(CO vs. CS); p = 0.000 defines p <0.001; significant p values are indicated in **bold text** (for $p<0.016$).

* for $p < 0.012$ for statistical significance in repeated measurements (differences between 'before induction' measurement and other individual measurements).

Table 5. Consumption of vasoactive/ inotropic drugs and insulin.

Measure	Group	Intraoperative	Postoperative		
			24 h after surgery	48 h after surgery	Postoperative day 5
Noradrenalin, total mg (median) [range]	MP	0.666 (1.3005) [0-6.27]	0.575 (2.675) [0-14.08]	0 (4.155) [0-37.98]	0 (0) [0-8.1]
	CS	0.2335 (0.4555) [0-2.147]	0.795 (2.83) [0-8.1]*	0 (1.8875) [0-12.2]	0 (0) [0-11.5]*
	CO	0.277 (0.399) [0-2.64]	0.285 (2.8475) [0-9.6]	0 (1.615) [0-7.4]	0 (0) [0-1.8]*
**p value**		0.024/0.047/0.714	0.978/0.880/0.902	0.733/0.276/0.455	0.384/0.132/0.554
Dobutamine, total mg (median) [range]	MP	0 (10.01) [0-146.08]	0 (9) [0-160]	0 (0) [0-580]	0 (0) [0-0]
	CS	1.315 (17.605) [0-78.67]	0 (36.625) [0-560]	0 (3.875) [0-675]	0 (0) [0-0]*
	CO	0 (10.915) [0-38.33]	0 (80.25) [0-350]*	0 (31.5) [0-600]*	0 (0) [0-0]*
**p value**		0.417/0.748/0.619	0.333/0.234/0.544	0.159/0.207/0.901	1.000/1.000/1.000
Levosimendan, total mg (median) [range]	MP	0 (0) [0-3.83]	0 (0) [0-3]	0 (0) [0-4]	0 (0) [0-0]
	CS	0 (0) [0-0]	0 (0) [0-0]	0 (0) [0-0]	0 (0) [0-0]
	CO	0 (0) [0-2.9]	0 (0) [0-7.65]	0 (0) [0-3.4]	0 (0) [0-0]
**p value**		0.317/0.594/0.152	0.317/0.264/0.076	0.317/0.337/0.076	1.000/1.000/1.000
Adrenalin, total µg (median) [range]	MP	0 (0) [0-0]	0 (0) [0-0]	0 (0) [0-0]	0 (0) [0-0]
	CS	0 (0) [0-25]	0 (0) [0-0]	0 (0) [0-0]	0 (0) [0-0]
	CO	0 (0) [0-0]	0 (0) [0-0]	0 (0) [0-0]	0 (0) [0-0]
**p value**		0.317/1.000/0.317	1.000/1.000/1.000	1.000/1.000/1.000	1.000/1.000/1.000
Nitronal, total mg (median) [range]	MP	0 (0) [0-3.23]	0 (29.025) [0-149]*	0 (94.35) [0-240]*	0 (0) [0-0]
	CS	0 (0) [0-13.2]	0 (28) [0-98]*	0 (33) [0-202]*	0 (0) [0-15]
	CO	0 (0) [0-35.82]	0 (27.75) [0-152]	0 (78.25) [0-256]	0 (0) [0-78]
**p value**		0.554/0.971/0.594	0.927/0.901/0.938	0.625/0.568/0.924	0.317/0.152/0.515
Insulin, total IU (median) [range]	MP		26 (36) [0-88]	35 (63.125) [0-206]	0 (0) [0-82]*
	CS		24 (9) [0-38]	14 (18.375) [0-121]*	0 (0) [0-22]*
	CO		22 (23) [0-56]	16 (27.5) [0-56]	0 (0) [0-133.5]*
**p value**			0.138/0.069/0.328	**0.000**/**0.003**/0.752	0.638/0.959/0.742

MP, Methylprednisolone; CS, Cytosorb; CO, Control.

p values- are shown for comparisons of groups at different time points: (MP vs. CS)/(MP vs. CO)/(CO vs. CS); p = 0.000 defines p <0.001; significant p values are indicated in **bold text** (for $p<0.016$).

* for $p < 0.016$ for statistical significance in repeated measurements (differences between 'before induction' measurement and other individual measurements).

Table 6. Correlations between inflammatory and biochemical parameters with four procedural times. Spearman's correlation coefficient (p value = 0.0001 after Bonferroni correction for multiple comparisons).

			Duration of anesthesia	CPB duration	Duration of surgery	Cross-clamp duration
Spearman's rho						
	TNFα1	Correlation Coefficient	0,065	0,191	0,115	0,081
		Sig. (2-tailed)	0,621	0,144	0,382	0,539
		N	60	60	60	60
	TNFα2	Correlation Coefficient	-0,070	-0,036	-0,055	-0,035
		Sig. (2-tailed)	0,595	0,786	0,678	0,789
		N	60	60	60	60
	TNFα3	Correlation Coefficient	-0,174	-0,179	-0,147	-0,126
		Sig. (2-tailed)	0,183	0,171	0,263	0,338
		N	60	60	60	60
	TNFα4	Correlation Coefficient	-0,041	-0,019	0,004	-0,035
		Sig. (2-tailed)	0,755	0,886	0,978	0,793
		N	60	60	60	60
	TNFα5	Correlation Coefficient	-0,028	0,018	0,018	-0,086
		Sig. (2-tailed)	0,830	0,891	0,891	0,512
		N	60	60	60	60
	TNFα6	Correlation Coefficient	0,131	0,005	0,136	-0,020
		Sig. (2-tailed)	0,318	0,973	0,300	0,881
		N	60	60	60	60
	IL-1 1.	Correlation Coefficient	-0,186	-0,157	-0,171	-0,196
		Sig. (2-tailed)	0,155	0,232	0,192	0,133
		N	60	60	60	60
	IL-1 2.	Correlation Coefficient	0,167	0,217	0,203	0,147
		Sig. (2-tailed)	0,203	0,095	0,119	0,263
		N	60	60	60	60

	IL-1 3.	Correlation Coefficient	0,243	0,169	,270*	0,162
		Sig. (2-tailed)	0,062	0,198	0,037	0,215
		N	60	60	60	60
	IL-1 4.	Correlation Coefficient	-0,019	0,018	0,055	0,062
		Sig. (2-tailed)	0,884	0,891	0,678	0,637
		N	60	60	60	60
	IL-1 5.	Correlation Coefficient	0,098	0,214	0,120	0,179
		Sig. (2-tailed)	0,455	0,100	0,361	0,172
		N	60	60	60	60
	IL-1 6.	Correlation Coefficient	-0,098	-0,074	-0,082	-0,181
		Sig. (2-tailed)	0,454	0,574	0,535	0,166
		N	60	60	60	60
	IL-6 1.	Correlation Coefficient	0,117	0,197	0,122	,309*
		Sig. (2-tailed)	0,371	0,132	0,352	0,016
		N	60	60	60	60
	IL-6 2.	Correlation Coefficient	,430**	,407**	,447**	,378**
		Sig. (2-tailed)	0,001	0,001	0,000	0,003
		N	60	60	60	60
	IL-6 3.	Correlation Coefficient	0,135	0,076	0,137	0,173
		Sig. (2-tailed)	0,305	0,563	0,297	0,187
		N	60	60	60	60
	IL-6 4.	Correlation Coefficient	-0,055	-0,106	-0,019	0,015
		Sig. (2-tailed)	0,679	0,422	0,885	0,907
		N	60	60	60	60
	IL-6 5.	Correlation Coefficient	-0,042	-0,071	0,015	-0,029
		Sig. (2-tailed)	0,751	0,592	0,910	0,824
		N	60	60	60	60
	IL-6 6.	Correlation Coefficient	0,034	0,083	0,113	0,023
		Sig. (2-tailed)	0,798	0,530	0,390	0,863
		N	60	60	60	60
	IL-8 1.	Correlation Coefficient	-0,037	0,052	-0,024	0,037
		Sig. (2-tailed)	0,777	0,692	0,856	0,777
		N	60	60	60	60

	IL-8 2.	Correlation Coefficient	,262*	0,178	,272*	0,128
		Sig. (2-tailed)	0,043	0,173	0,036	0,329
		N	60	60	60	60
	IL-8 3.	Correlation Coefficient	0,146	0,084	0,149	0,115
		Sig. (2-tailed)	0,266	0,524	0,255	0,383
		N	60	60	60	60
	IL-8 4.	Correlation Coefficient	0,058	0,145	0,121	0,170
		Sig. (2-tailed)	0,662	0,269	0,357	0,195
		N	60	60	60	60
	IL-8 5.	Correlation Coefficient	0,044	0,074	0,054	0,125
		Sig. (2-tailed)	0,736	0,572	0,682	0,341
		N	60	60	60	60
	IL-8 6.	Correlation Coefficient	0,143	-0,035	0,140	0,002
		Sig. (2-tailed)	0,275	0,791	0,285	0,989
		N	60	60	60	60
	IL-10 1.	Correlation Coefficient	-0,050	-0,015	-0,076	-0,001
		Sig. (2-tailed)	0,704	0,911	0,565	0,992
		N	60	60	60	60
	IL-10 2.	Correlation Coefficient	-0,072	-0,027	-0,093	-0,064
		Sig. (2-tailed)	0,582	0,839	0,481	0,626
		N	60	60	60	60
	IL-10 3.	Correlation Coefficient	-0,103	0,023	-0,109	0,008
		Sig. (2-tailed)	0,433	0,864	0,409	0,950
		N	60	60	60	60
	IL-10 4.	Correlation Coefficient	0,032	0,019	0,069	0,010
		Sig. (2-tailed)	0,809	0,883	0,602	0,939
		N	60	60	60	60
	IL-10 5.	Correlation Coefficient	0,076	0,141	0,071	0,102
		Sig. (2-tailed)	0,564	0,284	0,588	0,440
		N	60	60	60	60
	IL-10 6.	Correlation Coefficient	0,113	0,104	0,110	-0,001
		Sig. (2-tailed)	0,392	0,428	0,405	0,993
		N	60	60	60	60

	C5a 1.	Correlation Coefficient	-,295*	-,276*	-,332**	-,303*
		Sig. (2-tailed)	0,022	0,033	0,010	0,018
		N	60	60	60	60
	C5a 2.	Correlation Coefficient	-0,179	-0,130	-0,183	-0,121
		Sig. (2-tailed)	0,171	0,323	0,162	0,357
		N	60	60	60	60
	C5a 3.	Correlation Coefficient	0,107	0,141	0,147	0,074
		Sig. (2-tailed)	0,417	0,283	0,261	0,576
		N	60	60	60	60
	C5a 4.	Correlation Coefficient	-0,097	-0,148	-0,131	-0,054
		Sig. (2-tailed)	0,462	0,258	0,317	0,680
		N	60	60	60	60
	C5a 5.	Correlation Coefficient	-0,202	-0,169	-0,214	-0,150
		Sig. (2-tailed)	0,121	0,196	0,100	0,253
		N	60	60	60	60
	C5a 6.	Correlation Coefficient	-0,114	-0,157	-0,115	-0,248
		Sig. (2-tailed)	0,386	0,232	0,383	0,056
		N	60	60	60	60
	CD64 lim1	Correlation Coefficient	-0,063	-0,126	-0,105	-0,050
		Sig. (2-tailed)	0,635	0,336	0,426	0,704
		N	60	60	60	60
	CD64 lim2	Correlation Coefficient	0,016	-0,106	-0,034	0,003
		Sig. (2-tailed)	0,903	0,419	0,796	0,982
		N	60	60	60	60
	CD64 lim3	Correlation Coefficient	-0,036	-0,202	-0,080	-0,087
		Sig. (2-tailed)	0,785	0,122	0,545	0,509
		N	60	60	60	60
	CD64 lim4	Correlation Coefficient	-0,124	-0,172	-0,157	-0,106
		Sig. (2-tailed)	0,345	0,189	0,230	0,418
		N	60	60	60	60
	CD64 lim5	Correlation Coefficient	0,240	0,083	0,240	0,144
		Sig. (2-tailed)	0,064	0,527	0,065	0,274
		N	60	60	60	60

	CD64 lim6	Correlation Coefficient	-0,056	-0,161	-0,149	-0,133
		Sig. (2-tailed)	0,673	0,220	0,255	0,310
		N	60	60	60	60
	CD64mo1	Correlation Coefficient	0,026	-0,118	-0,021	-0,064
		Sig. (2-tailed)	0,841	0,368	0,874	0,629
		N	60	60	60	60
	CD64mo2	Correlation Coefficient	0,040	-0,018	0,037	0,029
		Sig. (2-tailed)	0,761	0,893	0,777	0,824
		N	60	60	60	60
	CD64mo3	Correlation Coefficient	0,154	0,084	0,148	0,086
		Sig. (2-tailed)	0,239	0,526	0,258	0,516
		N	60	60	60	60
	CD64mo4	Correlation Coefficient	-0,117	-0,119	-0,110	-0,015
		Sig. (2-tailed)	0,373	0,367	0,404	0,910
		N	60	60	60	60
	CD64mo5	Correlation Coefficient	-0,130	-0,168	-0,103	-0,083
		Sig. (2-tailed)	0,323	0,201	0,434	0,530
		N	60	60	60	60
	CD64mo6	Correlation Coefficient	-0,083	-0,044	-0,068	-0,105
		Sig. (2-tailed)	0,530	0,739	0,604	0,426
		N	60	60	60	60
	CD64gra1	Correlation Coefficient	-0,018	-0,125	-0,057	-0,140
		Sig. (2-tailed)	0,893	0,340	0,667	0,286
		N	60	60	60	60
	CD64gra2	Correlation Coefficient	0,147	0,021	0,097	-0,024
		Sig. (2-tailed)	0,262	0,871	0,461	0,853
		N	60	60	60	60
	CD64gra3	Correlation Coefficient	0,144	0,073	0,125	0,072
		Sig. (2-tailed)	0,273	0,580	0,342	0,587
		N	60	60	60	60
	CD64gra4	Correlation Coefficient	0,028	0,025	-0,010	-0,014
		Sig. (2-tailed)	0,831	0,849	0,937	0,914
		N	60	60	60	60

	CD64gra5	Correlation Coefficient	0,191	0,133	0,149	0,030
		Sig. (2-tailed)	0,144	0,309	0,255	0,822
		N	60	60	60	60
	CD64gra6	Correlation Coefficient	0,008	0,047	-0,043	0,001
		Sig. (2-tailed)	0,951	0,719	0,743	0,996
		N	60	60	60	60
	CD163lim1	Correlation Coefficient	0,060	-0,022	0,013	-0,044
		Sig. (2-tailed)	0,649	0,865	0,920	0,736
		N	60	60	60	60
	CD163lim2	Correlation Coefficient	0,004	-0,058	-0,040	-0,069
		Sig. (2-tailed)	0,978	0,662	0,763	0,601
		N	60	60	60	60
	CD163lim3	Correlation Coefficient	-0,065	-0,116	-0,097	-0,052
		Sig. (2-tailed)	0,621	0,378	0,461	0,693
		N	60	60	60	60
	CD163lim4	Correlation Coefficient	-0,040	-0,119	-0,064	-0,157
		Sig. (2-tailed)	0,761	0,365	0,628	0,232
		N	60	60	60	60
	CD163lim5	Correlation Coefficient	0,197	0,125	0,219	0,160
		Sig. (2-tailed)	0,132	0,343	0,093	0,223
		N	60	60	60	60
	CD163lim6	Correlation Coefficient	0,082	-0,033	-0,004	-0,108
		Sig. (2-tailed)	0,535	0,800	0,975	0,412
		N	60	60	60	60
	CD163mo1	Correlation Coefficient	-0,037	-0,167	-0,089	-0,225
		Sig. (2-tailed)	0,778	0,202	0,498	0,084
		N	60	60	60	60
	CD163mo2	Correlation Coefficient	-0,170	-0,126	-0,189	-0,051
		Sig. (2-tailed)	0,194	0,337	0,148	0,700
		N	60	60	60	60
	CD163mo3	Correlation Coefficient	0,213	,294*	0,208	0,178
		Sig. (2-tailed)	0,102	0,023	0,111	0,173
		N	60	60	60	60

	CD163mo4	Correlation Coefficient	0,167	0,228	0,147	0,104
		Sig. (2-tailed)	0,201	0,079	0,261	0,429
		N	60	60	60	60
	CD163mo5	Correlation Coefficient	0,199	,276*	0,172	0,194
		Sig. (2-tailed)	0,127	0,033	0,189	0,137
		N	60	60	60	60
	CD163mo6	Correlation Coefficient	-0,067	0,037	-0,106	0,001
		Sig. (2-tailed)	0,612	0,778	0,422	0,993
		N	60	60	60	60
	CD163gra1	Correlation Coefficient	-0,173	-0,107	-0,178	-0,127
		Sig. (2-tailed)	0,185	0,414	0,173	0,335
		N	60	60	60	60
	CD163gra2	Correlation Coefficient	-0,156	-0,110	-0,181	-0,141
		Sig. (2-tailed)	0,234	0,401	0,167	0,283
		N	60	60	60	60
	CD163gra3	Correlation Coefficient	-,271*	-0,209	-,291*	-0,185
		Sig. (2-tailed)	0,036	0,108	0,024	0,157
		N	60	60	60	60
	CD163gra4	Correlation Coefficient	0,058	0,096	0,033	0,017
		Sig. (2-tailed)	0,662	0,464	0,802	0,899
		N	60	60	60	60
	CD163gra5	Correlation Coefficient	0,187	0,197	0,166	0,103
		Sig. (2-tailed)	0,153	0,131	0,206	0,434
		N	60	60	60	60
	CD163gra6	Correlation Coefficient	0,101	0,026	0,003	0,014
		Sig. (2-tailed)	0,444	0,846	0,981	0,916
		N	60	60	60	60
	hs-CRP 1.	Correlation Coefficient	0,186	0,122	0,143	0,250
		Sig. (2-tailed)	0,154	0,353	0,277	0,054
		N	60	60	60	60
	hs-CRP 2.	Correlation Coefficient	0,164	0,194	0,121	,297*
		Sig. (2-tailed)	0,210	0,137	0,358	0,021
		N	60	60	60	60

	hs-CRP 3.	Correlation Coefficient	,302*	0,234	0,250	,316*
		Sig. (2-tailed)	0,019	0,071	0,054	0,014
		N	60	60	60	60
	hs-CRP 4.	Correlation Coefficient	0,022	0,088	0,072	0,194
		Sig. (2-tailed)	0,865	0,501	0,585	0,137
		N	60	60	60	60
	hs-CRP 5.	Correlation Coefficient	-0,004	-0,070	0,060	-0,006
		Sig. (2-tailed)	0,977	0,595	0,648	0,963
		N	60	60	60	60
	hs-CRP 6.	Correlation Coefficient	0,071	0,127	0,152	0,154
		Sig. (2-tailed)	0,592	0,335	0,246	0,239
		N	60	60	60	60
	PCT 1.	Correlation Coefficient	0,113	0,000	0,071	0,031
		Sig. (2-tailed)	0,388	0,997	0,588	0,816
		N	60	60	60	60
	PCT 2.	Correlation Coefficient	0,225	0,175	0,215	0,195
		Sig. (2-tailed)	0,084	0,182	0,099	0,136
		N	60	60	60	60
	PCT 3.	Correlation Coefficient	,381**	,301*	,373**	0,246
		Sig. (2-tailed)	0,003	0,020	0,003	0,058
		N	60	60	60	60
	PCT 4.	Correlation Coefficient	0,092	0,094	0,119	0,135
		Sig. (2-tailed)	0,486	0,477	0,364	0,305
		N	60	60	60	60
	PCT 5.	Correlation Coefficient	0,177	0,136	0,199	0,199
		Sig. (2-tailed)	0,176	0,301	0,128	0,127
		N	60	60	60	60
	PCT 6.	Correlation Coefficient	0,217	0,213	0,222	0,178
		Sig. (2-tailed)	0,096	0,103	0,088	0,173
		N	60	60	60	60
	Le 1.	Correlation Coefficient	-0,011	-0,008	-0,037	0,129
		Sig. (2-tailed)	0,933	0,950	0,782	0,326
		N	60	60	60	60

	Le 2.	Correlation Coefficient	0,135	0,071	0,109	0,125
		Sig. (2-tailed)	0,307	0,595	0,410	0,345
		N	59	59	59	59
	Le 3.	Correlation Coefficient	0,071	0,126	0,075	0,215
		Sig. (2-tailed)	0,592	0,336	0,570	0,099
		N	60	60	60	60
	Le 4.	Correlation Coefficient	0,030	,283*	0,077	,287*
		Sig. (2-tailed)	0,823	0,030	0,563	0,028
		N	59	59	59	59
	Le 5.	Correlation Coefficient	-0,052	0,063	-0,021	0,125
		Sig. (2-tailed)	0,692	0,634	0,876	0,343
		N	60	60	60	60
	Le 6.	Correlation Coefficient	-0,122	-0,032	-0,143	0,014
		Sig. (2-tailed)	0,354	0,809	0,276	0,916
		N	60	60	60	60
	Albumin 1.	Correlation Coefficient	-0,154	-0,137	-0,126	-0,156
		Sig. (2-tailed)	0,241	0,296	0,339	0,233
		N	60	60	60	60
	Albumin 2.	Correlation Coefficient	-0,042	0,125	-0,047	-0,014
		Sig. (2-tailed)	0,748	0,339	0,724	0,915
		N	60	60	60	60
	Albumin 3.	Correlation Coefficient	0,102	0,147	0,060	0,017
		Sig. (2-tailed)	0,437	0,261	0,647	0,900
		N	60	60	60	60
	Albumin 4.	Correlation Coefficient	-,273*	-0,092	-,256*	-0,176
		Sig. (2-tailed)	0,035	0,482	0,048	0,178
		N	60	60	60	60
	Albumin 5.	Correlation Coefficient	-0,196	-0,121	-0,234	-0,178
		Sig. (2-tailed)	0,133	0,359	0,072	0,173
		N	60	60	60	60
	Albumin 6.	Correlation Coefficient	-,286*	-,328*	-,361**	-,427**
		Sig. (2-tailed)	0,027	0,010	0,005	0,001
		N	60	60	60	60

	Fibrinog.1.	Correlation Coefficient	0,040	-0,073	-0,006	0,069
		Sig. (2-tailed)	0,760	0,578	0,964	0,600
		N	60	60	60	60
	Fibrinog.2.	Correlation Coefficient	0,133	0,092	0,063	0,183
		Sig. (2-tailed)	0,314	0,488	0,635	0,165
		N	59	59	59	59
	Fibrinog.3.	Correlation Coefficient	,329*	0,167	0,246	0,191
		Sig. (2-tailed)	0,010	0,202	0,058	0,143
		N	60	60	60	60
	Fibrinog.4.	Correlation Coefficient	0,126	0,074	0,074	0,127
		Sig. (2-tailed)	0,338	0,575	0,575	0,335
		N	60	60	60	60
	Fibrinog.5.	Correlation Coefficient	-0,014	-0,149	-0,042	-0,113
		Sig. (2-tailed)	0,915	0,256	0,750	0,389
		N	60	60	60	60
	Fibrinog.6.	Correlation Coefficient	-0,083	-0,225	-0,101	-0,241
		Sig. (2-tailed)	0,531	0,084	0,445	0,063
		N	60	60	60	60
	Hb 1	Correlation Coefficient	-0,047	-0,099	-0,082	-0,174
		Sig. (2-tailed)	0,722	0,453	0,535	0,184
		N	60	60	60	60
	Hb 2	Correlation Coefficient	-0,159	-0,099	-0,187	-0,171
		Sig. (2-tailed)	0,229	0,455	0,156	0,196
		N	59	59	59	59
	Hb 3	Correlation Coefficient	-0,099	-0,011	-0,124	-0,063
		Sig. (2-tailed)	0,455	0,935	0,349	0,633
		N	59	59	59	59
	Hb 4	Correlation Coefficient	-0,255	-0,052	-0,236	-0,032
		Sig. (2-tailed)	0,051	0,694	0,072	0,812
		N	59	59	59	59
	Hb 5	Correlation Coefficient	-0,174	-0,147	-0,186	-0,151
		Sig. (2-tailed)	0,183	0,263	0,154	0,248
		N	60	60	60	60

	Hb 6	Correlation Coefficient	-0,051	0,097	-0,055	0,031
		Sig. (2-tailed)	0,700	0,461	0,678	0,814
		N	60	60	60	60
	PLT 1	Correlation Coefficient	-0,071	-0,064	-0,023	0,027
		Sig. (2-tailed)	0,591	0,630	0,859	0,838
		N	60	60	60	60
	PLT 2	Correlation Coefficient	-0,082	-0,070	-0,095	-0,109
		Sig. (2-tailed)	0,543	0,601	0,479	0,415
		N	58	58	58	58
	PLT 3	Correlation Coefficient	-0,013	-0,050	-0,008	-0,013
		Sig. (2-tailed)	0,922	0,710	0,955	0,920
		N	58	58	58	58
	PLT 4	Correlation Coefficient	-0,187	-0,039	-0,129	-0,089
		Sig. (2-tailed)	0,153	0,766	0,327	0,500
		N	60	60	60	60
	PLT 5	Correlation Coefficient	-0,197	-0,089	-0,152	-0,075
		Sig. (2-tailed)	0,131	0,500	0,247	0,567
		N	60	60	60	60
	PLT 6	Correlation Coefficient	-0,152	-0,218	-0,153	-,262*
		Sig. (2-tailed)	0,252	0,098	0,246	0,045
		N	59	59	59	59

Table 7. Kruskal Wallis test for testing the association between inflammatory mediators and biochemical parameters with eight different types of surgical procedures (p value = 0.0018 after Bonferroni correction for multiple comparisons).

	Kruskal-Wallis H	df	Asymp. Sig.
TNFα1	10,101	7	0,183
TNFα2	5,614	7	0,585
TNFα3	8,530	7	0,288
TNFα4	2,907	7	0,894
TNFα5	7,594	7	0,370
TNFα6	3,684	7	0,815
IL-1 1.	4,573	7	0,712
IL-1 2.	9,423	7	0,224
IL-1 3.	7,986	7	0,334
IL-1 4.	5,717	7	0,573
IL-1 5.	8,662	7	0,278
IL-1 6.	15,930	7	0,026
IL-6 1.	16,253	7	0,023
IL-6 2.	7,171	7	0,411
IL-6 3.	5,166	7	0,640
IL-6 4.	5,618	7	0,585
IL-6 5.	6,405	7	0,493
IL-6 6.	10,713	7	0,152
IL-8 1.	3,130	7	0,873
IL-8 2.	3,953	7	0,785
IL-8 3.	1,972	7	0,961
IL-8 4.	10,041	7	0,186
IL-8 5.	6,020	7	0,537
IL-8 6.	3,305	7	0,855
IL-10 1.	5,768	7	0,567
IL-10 2.	6,539	7	0,478
IL-10 3.	8,368	7	0,301

IL-10 4.	8,157	7	0,319
IL-10 5.	3,065	7	0,879
IL-10 6.	6,560	7	0,476
C5a 1.	10,155	7	0,180
C5a 2.	7,508	7	0,378
C5a 3.	6,978	7	0,431
C5a 4.	5,345	7	0,618
C5a 5.	6,991	7	0,430
C5a 6.	3,265	7	0,859
hs-CRP 1.	6,041	7	0,535
hs-CRP 2.	6,460	7	0,487
hs-CRP 3.	7,586	7	0,371
hs-CRP 4.	10,146	7	0,180
hs-CRP 5.	9,842	7	0,198
Le 4.	4,606	7	0,708
Le 5.	7,456	7	0,383
Le 6.	7,261	7	0,402
Albumin 1.	5,847	7	0,558
Albumin 2.	6,974	7	0,432
Albumin 3.	11,948	7	0,102
Albumin 4.	13,669	7	0,057
Albumin 5.	12,877	7	0,075
Albumin 6.	14,608	7	0,041
Fibrinog.1.	9,573	7	0,214
Fibrinog.2.	7,351	7	0,393
Fibrinog.3.	12,095	7	0,097
Fibrinog.4.	7,175	7	0,411
Fibrinog.5.	16,472	7	0,021
Fibrinog.6.	18,073	7	0,012
CD64 lim1	13,704	7	0,057
CD64 lim2	6,798	7	0,450
CD64 lim3	8,600	7	0,283
CD64 lim4	6,827	7	0,447

CD64 lim5	6,664	7	0,465
CD64 lim6	6,354	7	0,499
CD64mo1	11,022	7	0,138
CD64mo2	7,395	7	0,389
CD64mo3	5,487	7	0,601
CD64mo4	4,382	7	0,735
CD64mo5	6,620	7	0,469
CD64mo6	10,654	7	0,154
CD64gra1	17,562	7	0,014
CD64gra2	7,466	7	0,382
CD64gra3	1,879	7	0,966
CD64gra4	6,130	7	0,525
CD64gra5	6,119	7	0,526
CD64gra6	10,947	7	0,141
CD163lim1	5,529	7	0,596
CD163lim2	10,208	7	0,177
CD163lim3	4,578	7	0,711
CD163lim4	9,462	7	0,221
CD163lim5	6,409	7	0,493
CD163lim6	5,335	7	0,619
CD163mo1	12,269	7	0,092
CD163mo2	6,686	7	0,462
CD163mo3	7,017	7	0,427
CD163mo4	5,408	7	0,610
CD163mo5	5,790	7	0,564
CD163mo6	5,575	7	0,590
CD163gra1	5,846	7	0,558
CD163gra2	10,922	7	0,142
CD163gra3	4,735	7	0,692
CD163gra4	4,578	7	0,711
CD163gra5	4,537	7	0,716
CD163gra6	8,254	7	0,311
Hb 1	6,501	7	0,483

Hb 2	9,560	7	0,215
Hb 3	7,470	7	0,382
Hb 4	10,546	7	0,160
Hb 5	16,271	7	0,023
Hb 6	12,022	7	0,100
PLT 1	9,980	7	0,190
PLT 2	3,482	7	0,837
PLT 3	12,625	7	0,082
PLT 4	17,915	7	0,012
PLT 5	17,948	7	0,012
PLT 6	20,657	7	0,004

Coding of different types of surgical procedures:

1- One valve surgery + CABG

2 - More than one valve surgery + CABG

3 - More than one valve surgery

4 - Surgery of ascending aorta

5 - Valve surgery + CABG + surgery of ascending aorta

6 - Valve surgery + surgery of ascending aorta

7 - Valve surgery + other procedures (ASD, RFA)

8 - Valve surgery + surgery of ascending aorta + other procedure (ASD, RFA)

Table 8. Range values in groups of different surgical procedures.

Ranks

surgical procedure 1-8		N	Mean Rank
TNFα1	1	20	30,15
	2	3	45,50
	3	13	28,62
	4	11	27,05
	5	2	9,00
	6	8	35,75
	7	2	51,00
	8	1	15,00
	Total	60	
TNFα2	1	20	29,40
	2	3	40,17
	3	13	27,69
	4	11	31,41
	5	2	16,00
	6	8	34,88
	7	2	45,50
	8	1	14,00
	Total	60	
TNFα3	1	20	30,50
	2	3	44,00
	3	13	22,85
	4	11	36,45
	5	2	21,00
	6	8	36,00
	7	2	25,00
	8	1	10,00
	Total	60	
TNFα4	1	20	30,60
	2	3	25,33

	3	13	27,04
	4	11	33,82
	5	2	33,00
	6	8	34,44
	7	2	33,00
	8	1	11,00
	Total	60	
TNF5	1	20	30,35
	2	3	39,33
	3	13	22,85
	4	11	28,82
	5	2	32,50
	6	8	35,25
	7	2	42,50
	8	1	59,00
	Total	60	
TNFα6	1	20	33,83
	2	3	39,00
	3	13	29,81
	4	11	26,18
	5	2	31,50
	6	8	30,38
	7	2	20,50
	8	1	14,00
	Total	60	
IL-1 1.	1	20	29,30
	2	3	26,67
	3	13	25,35
	4	11	30,95
	5	2	28,50
	6	8	40,63
	7	2	38,00
	8	1	36,00

	Total	60	
IL-1 2.	1	20	25,75
	2	3	46,83
	3	13	33,65
	4	11	28,36
	5	2	21,00
	6	8	30,63
	7	2	55,50
	8	1	27,00
	Total	60	
IL-1 3.	1	20	26,28
	2	3	31,67
	3	13	34,38
	4	11	25,05
	5	2	54,50
	6	8	36,25
	7	2	34,00
	8	1	20,00
	Total	60	
IL-1 4.	1	20	27,40
	2	3	22,33
	3	13	31,92
	4	11	29,45
	5	2	54,50
	6	8	34,63
	7	2	32,00
	8	1	26,00
	Total	60	
IL-1 5.	1	20	27,50
	2	3	42,67
	3	13	32,38
	4	11	23,36
	5	2	39,00

	6	8	34,00
	7	2	53,00
	8	1	18,00
	Total	60	
IL-1 6.	1	20	24,85
	2	3	42,00
	3	13	27,81
	4	11	24,41
	5	2	26,00
	6	8	47,00
	7	2	49,50
	8	1	50,00
	Total	60	
IL-6 1.	1	20	35,85
	2	3	48,67
	3	13	24,69
	4	11	15,91
	5	2	30,50
	6	8	38,63
	7	2	36,50
	8	1	28,00
	Total	60	
IL-6 2.	1	20	25,80
	2	3	47,67
	3	13	36,27
	4	11	25,45
	5	2	31,50
	6	8	31,81
	7	2	38,00
	8	1	26,00
	Total	60	
IL-6 3.	1	20	27,15
	2	3	36,33

	3	13	36,38
	4	11	27,77
	5	2	32,50
	6	8	34,81
	7	2	23,50
	8	1	9,00
	Total	60	
IL-6 4.	1	20	26,83
	2	3	29,33
	3	13	31,00
	4	11	33,36
	5	2	41,00
	6	8	37,06
	7	2	27,00
	8	1	3,00
	Total	60	
IL-6 5.	1	20	27,98
	2	3	32,33
	3	13	26,38
	4	11	32,23
	5	2	36,50
	6	8	40,75
	7	2	35,50
	8	1	6,00
	Total	60	
IL-6 6.	1	20	26,83
	2	3	48,00
	3	13	21,92
	4	11	34,23
	5	2	31,50
	6	8	37,75
	7	2	47,00
	8	1	29,00

	Total	60	
IL-8 1.	1	20	33,30
	2	3	36,50
	3	13	26,77
	4	11	25,50
	5	2	26,75
	6	8	34,38
	7	2	36,00
	8	1	25,50
	Total	60	
IL-8 2.	1	20	30,33
	2	3	29,00
	3	13	35,00
	4	11	26,45
	5	2	26,75
	6	8	28,00
	7	2	47,00
	8	1	19,00
	Total	60	
IL-8 3.	1	20	29,73
	2	3	32,67
	3	13	34,46
	4	11	29,09
	5	2	24,75
	6	8	31,75
	7	2	25,00
	8	1	16,00
	Total	60	
IL-8 4.	1	20	31,70
	2	3	26,67
	3	13	29,08
	4	11	24,45
	5	2	58,00

	6	8	33,50
	7	2	41,00
	8	1	3,00
	Total	60	
IL-8 5.	1	20	32,23
	2	3	31,50
	3	13	30,19
	4	11	23,82
	5	2	47,50
	6	8	26,31
	7	2	41,00
	8	1	49,00
	Total	60	
IL-8 6.	1	20	33,20
	2	3	31,33
	3	13	29,85
	4	11	25,45
	5	2	38,50
	6	8	32,56
	7	2	28,00
	8	1	10,50
	Total	60	
IL-10 1.	1	20	30,28
	2	3	41,33
	3	13	30,92
	4	11	24,14
	5	2	14,75
	6	8	36,13
	7	2	40,50
	8	1	33,50
	Total	60	
IL-10 2.	1	20	30,03
	2	3	39,00

	3	13	26,00
	4	11	24,95
	5	2	32,50
	6	8	37,88
	7	2	38,50
	8	1	55,00
	Total	60	
IL-10 3.	1	20	30,50
	2	3	42,00
	3	13	24,69
	4	11	24,32
	5	2	41,50
	6	8	34,88
	7	2	47,75
	8	1	48,00
	Total	60	
IL-10 4.	1	20	32,58
	2	3	25,33
	3	13	28,58
	4	11	29,27
	5	2	41,00
	6	8	26,38
	7	2	55,50
	8	1	5,00
	Total	60	
IL-10 5.	1	20	28,85
	2	3	39,00
	3	13	29,54
	4	11	30,55
	5	2	30,50
	6	8	31,31
	7	2	44,75
	8	1	15,00

	Total	60	
IL-10 6.	1	20	30,40
	2	3	42,00
	3	13	30,00
	4	11	26,27
	5	2	19,50
	6	8	30,88
	7	2	54,00
	8	1	23,00
	Total	60	
C5a 1.	1	20	30,68
	2	3	19,67
	3	13	20,73
	4	11	37,73
	5	2	45,00
	6	8	31,63
	7	2	42,50
	8	1	45,00
	Total	60	
C5a 2.	1	20	36,05
	2	3	10,67
	3	13	28,15
	4	11	27,55
	5	2	42,50
	6	8	29,00
	7	2	28,50
	8	1	34,00
	Total	60	
C5a 3.	1	20	31,73
	2	3	11,50
	3	13	27,38
	4	11	37,55
	5	2	24,00

	6	8	30,38
	7	2	27,50
	8	1	46,00
	Total	60	
C5a 4.	1	20	29,00
	2	3	15,33
	3	13	32,85
	4	11	36,18
	5	2	38,50
	6	8	26,38
	7	2	24,50
	8	1	42,00
	Total	60	
C5a 5.	1	20	25,35
	2	3	31,33
	3	13	32,92
	4	11	40,00
	5	2	26,00
	6	8	29,50
	7	2	16,50
	8	1	40,00
	Total	60	
C5a 6.	1	20	34,10
	2	3	24,67
	3	13	24,54
	4	11	32,73
	5	2	33,00
	6	8	28,13
	7	2	33,50
	8	1	37,00
	Total	60	
hs-CRP 1.	1	20	32,55
	2	3	31,00

	3	13	34,46
	4	11	20,09
	5	2	31,00
	6	8	35,13
	7	2	28,50
	8	1	17,00
	Total	60	
hs-CRP 2.	1	20	28,60
	2	3	36,00
	3	13	35,73
	4	11	21,18
	5	2	34,00
	6	8	37,56
	7	2	31,00
	8	1	22,00
	Total	60	
hs-CRP 3.	1	20	31,60
	2	3	38,67
	3	13	34,88
	4	11	18,86
	5	2	29,50
	6	8	34,88
	7	2	33,00
	8	1	17,00
	Total	60	
hs-CRP 4.	1	20	32,95
	2	3	10,00
	3	13	31,08
	4	11	37,09
	5	2	35,75
	6	8	27,75
	7	2	15,25
	8	1	5,00

	Total	60	
hs-CRP 5.	1	20	28,90
	2	3	10,33
	3	13	31,23
	4	11	39,41
	5	2	29,25
	6	8	34,38
	7	2	20,00
	8	1	8,00
	Total	60	
hs-CRP 6.	1	20	32,05
	2	3	22,50
	3	13	25,81
	4	11	39,09
	5	2	31,50
	6	8	32,13
	7	2	15,00
	8	1	6,00
	Total	60	
PCT 1.	1	20	32,75
	2	3	38,00
	3	13	35,12
	4	11	22,14
	5	2	20,25
	6	8	27,13
	7	2	31,50
	8	1	40,50
	Total	60	
PCT 2.	1	20	30,68
	2	3	36,67
	3	13	38,12
	4	11	30,00
	5	2	22,25

	6	8	22,13
	7	2	14,50
	8	1	30,50
	Total	60	
PCT 3.	1	20	29,45
	2	3	47,67
	3	13	36,77
	4	11	30,68
	5	2	20,25
	6	8	22,06
	7	2	19,25
	8	1	27,00
	Total	60	
PCT 4.	1	20	28,90
	2	3	40,83
	3	13	34,27
	4	11	28,36
	5	2	20,50
	6	8	33,63
	7	2	26,00
	8	1	10,00
	Total	60	
PCT 5.	1	20	29,43
	2	3	41,50
	3	13	35,46
	4	11	27,59
	5	2	23,75
	6	8	30,69
	7	2	24,25
	8	1	11,00
	Total	60	
PCT 6.	1	20	30,80
	2	3	38,17

	3	13	33,23
	4	11	24,41
	5	2	6,25
	6	8	36,19
	7	2	45,00
	8	1	7,00
	Total	60	
Le 1.	1	20	34,55
	2	3	30,00
	3	13	23,12
	4	11	29,05
	5	2	55,00
	6	8	35,13
	7	2	17,50
	8	1	3,00
	Total	60	
Le 2.	1	20	34,28
	2	3	43,33
	3	12	29,54
	4	11	19,50
	5	2	27,50
	6	8	29,81
	7	2	35,00
	8	1	22,00
	Total	59	
Le 3.	1	20	36,80
	2	3	37,17
	3	13	24,54
	4	11	30,05
	5	2	41,00
	6	8	21,25
	7	2	39,00
	8	1	3,00

	Total	60	
Le 4.	1	20	34,15
	2	3	24,50
	3	12	30,88
	4	11	28,95
	5	2	30,50
	6	8	26,69
	7	2	23,50
	8	1	3,00
	Total	59	
Le 5.	1	20	35,90
	2	3	25,33
	3	13	24,58
	4	11	35,00
	5	2	28,00
	6	8	28,44
	7	2	22,50
	8	1	3,00
	Total	60	
Le 6.	1	20	37,23
	2	3	31,83
	3	13	22,19
	4	11	30,23
	5	2	30,25
	6	8	31,44
	7	2	18,50
	8	1	20,00
	Total	60	
Albumin 1.	1	20	32,80
	2	3	28,67
	3	13	25,50
	4	11	36,86
	5	2	33,50

	6	8	30,06
	7	2	18,25
	8	1	7,00
	Total	60	
Albumin 2.	1	20	25,23
	2	3	43,83
	3	13	29,00
	4	11	29,23
	5	2	31,75
	6	8	37,31
	7	2	44,50
	8	1	44,50
	Total	60	
Albumin 3.	1	20	21,18
	2	3	48,00
	3	13	30,62
	4	11	33,73
	5	2	37,25
	6	8	37,38
	7	2	36,25
	8	1	47,50
	Total	60	
Albumin 4.	1	20	20,78
	2	3	42,17
	3	13	30,46
	4	11	39,36
	5	2	32,25
	6	8	38,25
	7	2	20,75
	8	1	47,00
	Total	60	
Albumin 5.	1	20	22,60
	2	3	42,83

	3	13	25,96
	4	11	40,41
	5	2	33,00
	6	8	37,94
	7	2	25,00
	8	1	48,00
	Total	60	
Albumin 6.	1	20	23,63
	2	3	16,17
	3	13	31,00
	4	11	44,64
	5	2	29,75
	6	8	32,31
	7	2	22,25
	8	1	52,50
	Total	60	
Fibrinog.1.	1	20	36,60
	2	3	37,33
	3	13	30,58
	4	11	22,95
	5	2	46,00
	6	8	24,56
	7	2	15,75
	8	1	16,00
	Total	60	
Fibrinog.2.	1	20	30,65
	2	3	46,00
	3	12	32,04
	4	11	20,86
	5	2	38,75
	6	8	27,06
	7	2	34,00
	8	1	43,00

	Total	59	
Fibrinog.3.	1	20	27,90
	2	3	41,33
	3	13	40,58
	4	11	21,77
	5	2	36,75
	6	8	22,88
	7	2	46,25
	8	1	32,00
	Total	60	
Fibrinog.4.	1	20	26,73
	2	3	28,17
	3	13	36,08
	4	11	32,00
	5	2	49,00
	6	8	30,63
	7	2	20,00
	8	1	7,00
	Total	60	
Fibrinog.5.	1	20	23,98
	2	3	23,17
	3	13	31,96
	4	11	44,64
	5	2	47,25
	6	8	31,13
	7	2	10,50
	8	1	10,00
	Total	60	
Fibrinog.6.	1	20	26,40
	2	3	17,83
	3	13	25,00
	4	11	45,55
	5	2	55,50

	6	8	32,44
	7	2	16,00
	8	1	20,00
	Total	60	
CD64 lim1	1	20	24,93
	2	3	47,17
	3	13	36,77
	4	11	19,50
	5	2	42,00
	6	8	38,63
	7	2	32,50
	8	1	39,50
	Total	60	
CD64 lim2	1	20	27,18
	2	3	37,67
	3	13	31,81
	4	11	26,05
	5	2	54,50
	6	8	35,44
	7	2	30,50
	8	1	20,00
	Total	60	
CD64 lim3	1	20	26,78
	2	3	46,83
	3	13	30,31
	4	11	27,18
	5	2	56,50
	6	8	32,75
	7	2	30,50
	8	1	25,00
	Total	60	
CD64 lim4	1	20	27,00
	2	3	40,67

	3	13	26,96
	4	11	33,82
	5	2	49,50
	6	8	31,94
	7	2	39,50
	8	1	12,00
	Total	60	
CD64 lim5	1	20	26,88
	2	3	38,33
	3	13	32,00
	4	11	33,95
	5	2	49,00
	6	8	28,81
	7	2	27,75
	8	1	4,00
	Total	60	
CD64 lim6	1	20	25,93
	2	3	32,17
	3	13	27,85
	4	11	33,09
	5	2	52,50
	6	8	36,81
	7	2	26,75
	8	1	36,00
	Total	60	
CD64mo1	1	20	26,10
	2	3	47,67
	3	13	32,77
	4	11	21,64
	5	2	31,00
	6	8	41,00
	7	2	32,00
	8	1	47,00

	Total	60	
CD64mo2	1	20	28,55
	2	3	43,33
	3	13	29,77
	4	11	22,18
	5	2	36,50
	6	8	39,75
	7	2	32,00
	8	1	43,00
	Total	60	
CD64mo3	1	20	25,80
	2	3	39,00
	3	13	32,15
	4	11	26,18
	5	2	37,00
	6	8	39,13
	7	2	32,00
	8	1	40,00
	Total	60	
CD64mo4	1	20	26,65
	2	3	24,67
	3	13	32,69
	4	11	31,00
	5	2	45,50
	6	8	36,50
	7	2	27,00
	8	1	20,00
	Total	60	
CD64mo5	1	20	26,60
	2	3	19,67
	3	13	30,62
	4	11	32,55
	5	2	37,00

	6	8	42,00
	7	2	27,50
	8	1	18,00
	Total	60	
CD64mo6	1	20	24,85
	2	3	26,00
	3	13	26,77
	4	11	33,55
	5	2	49,00
	6	8	44,25
	7	2	25,50
	8	1	35,00
	Total	60	
CD64gra1	1	20	25,25
	2	3	50,33
	3	13	28,54
	4	11	21,09
	5	2	35,50
	6	8	43,75
	7	2	49,00
	8	1	52,00
	Total	60	
CD64gra2	1	20	25,63
	2	3	38,33
	3	13	31,27
	4	11	25,86
	5	2	46,50
	6	8	37,38
	7	2	33,75
	8	1	52,00
	Total	60	
CD64gra3	1	20	28,15
	2	3	35,67

	3	13	31,15
	4	11	27,64
	5	2	34,50
	6	8	34,00
	7	2	34,00
	8	1	42,00
	Total	60	
CD64gra4	1	20	26,80
	2	3	29,00
	3	13	27,15
	4	11	32,36
	5	2	50,00
	6	8	34,00
	7	2	47,00
	8	1	32,00
	Total	60	
CD64gra5	1	20	25,20
	2	3	29,33
	3	13	28,46
	4	11	33,18
	5	2	37,00
	6	8	36,00
	7	2	45,00
	8	1	51,00
	Total	60	
CD64gra6	1	20	26,73
	2	3	27,00
	3	13	26,31
	4	11	26,55
	5	2	45,00
	6	8	45,19
	7	2	42,50
	8	1	44,00

	Total	60	
CD163lim1	1	20	27,38
	2	3	23,67
	3	13	35,12
	4	11	24,82
	5	2	40,75
	6	8	38,44
	7	2	31,50
	8	1	30,00
	Total	60	
CD163lim2	1	20	26,15
	2	3	38,17
	3	13	31,58
	4	11	24,32
	5	2	47,25
	6	8	36,94
	7	2	53,50
	8	1	17,50
	Total	60	
CD163lim3	1	20	28,73
	2	3	40,17
	3	13	29,65
	4	11	25,95
	5	2	47,00
	6	8	34,75
	7	2	35,25
	8	1	21,50
	Total	60	
CD163lim4	1	20	25,83
	2	3	43,33
	3	13	30,58
	4	11	39,86
	5	2	36,50

	6	8	23,69
	7	2	37,75
	8	1	9,50
	Total	60	
CD163lim5	1	20	35,05
	2	3	42,83
	3	13	29,12
	4	11	25,36
	5	2	37,00
	6	8	25,81
	7	2	27,00
	8	1	8,50
	Total	60	
CD163lim6	1	20	25,98
	2	3	44,00
	3	13	29,69
	4	11	33,18
	5	2	47,00
	6	8	29,69
	7	2	30,00
	8	1	36,00
	Total	60	
CD163mo1	1	20	24,20
	2	3	9,33
	3	13	33,77
	4	11	34,00
	5	2	44,00
	6	8	35,88
	7	2	37,00
	8	1	56,00
	Total	60	
CD163mo2	1	20	30,40
	2	3	23,00

	3	13	25,85
	4	11	26,91
	5	2	42,50
	6	8	37,25
	7	2	42,50
	8	1	53,00
	Total	60	
CD163mo3	1	20	29,85
	2	3	35,00
	3	13	29,85
	4	11	23,00
	5	2	30,00
	6	8	34,00
	7	2	52,50
	8	1	50,00
	Total	60	
CD163mo4	1	20	29,80
	2	3	39,33
	3	13	31,00
	4	11	24,91
	5	2	36,00
	6	8	27,88
	7	2	46,00
	8	1	52,00
	Total	60	
CD163mo5	1	20	32,15
	2	3	38,00
	3	13	31,04
	4	11	21,36
	5	2	32,50
	6	8	30,44
	7	2	36,00
	8	1	54,00

	Total	60	
CD163mo6	1	20	31,40
	2	3	13,67
	3	13	34,00
	4	11	25,55
	5	2	41,50
	6	8	30,25
	7	2	36,50
	8	1	40,00
	Total	60	
CD163gra1	1	20	30,28
	2	3	25,83
	3	13	31,54
	4	11	22,00
	5	2	31,50
	6	8	40,69
	7	2	35,25
	8	1	36,00
	Total	60	
CD163gra2	1	20	23,68
	2	3	22,17
	3	13	30,88
	4	11	31,95
	5	2	52,00
	6	8	41,56
	7	2	27,25
	8	1	46,00
	Total	60	
CD163gra3	1	20	28,03
	2	3	20,00
	3	13	31,92
	4	11	29,18
	5	2	49,50

	6	8	34,75
	7	2	28,75
	8	1	39,00
	Total	60	
CD163gra4	1	20	32,40
	2	3	33,33
	3	13	26,27
	4	11	25,68
	5	2	41,00
	6	8	30,88
	7	2	38,50
	8	1	52,00
	Total	60	
CD163gra5	1	20	32,28
	2	3	34,17
	3	13	29,04
	4	11	24,23
	5	2	41,75
	6	8	29,50
	7	2	32,25
	8	1	54,00
	Total	60	
CD163gra6	1	20	28,08
	2	3	33,33
	3	13	34,46
	4	11	21,95
	5	2	46,00
	6	8	34,75
	7	2	26,00
	8	1	57,00
	Total	60	
Hb 1	1	20	27,40
	2	3	23,17

	3	13	28,77
	4	11	39,14
	5	2	21,50
	6	8	36,19
	7	2	31,75
	8	1	12,00
	Total	60	
Hb 2	1	20	22,65
	2	3	38,83
	3	12	29,92
	4	11	36,91
	5	2	18,00
	6	8	38,44
	7	2	26,25
	8	1	39,50
	Total	59	
Hb 3	1	19	25,37
	2	3	26,33
	3	13	27,58
	4	11	36,41
	5	2	32,75
	6	8	35,13
	7	2	47,00
	8	1	9,50
	Total	59	
Hb 4	1	20	24,05
	2	3	18,33
	3	12	29,33
	4	11	40,32
	5	2	28,00
	6	8	39,06
	7	2	25,00
	8	1	20,00

	Total	59	
Hb 5	1	20	22,03
	2	3	25,00
	3	13	31,04
	4	11	41,64
	5	2	22,75
	6	8	43,19
	7	2	13,00
	8	1	36,00
	Total	60	
Hb 6	1	20	23,85
	2	3	19,83
	3	13	31,96
	4	11	42,86
	5	2	14,50
	6	8	33,94
	7	2	32,50
	8	1	41,00
	Total	60	
PLT 1	1	20	33,70
	2	3	17,17
	3	13	27,27
	4	11	38,14
	5	2	41,75
	6	8	26,31
	7	2	16,75
	8	1	3,00
	Total	60	
PLT 2	1	20	29,90
	2	3	29,00
	3	11	26,59
	4	11	36,73
	5	2	27,50

	6	8	25,13
	7	2	28,25
	8	1	17,00
	Total	58	
PLT 3	1	19	30,55
	2	3	24,50
	3	12	22,58
	4	11	41,59
	5	2	43,25
	6	8	24,06
	7	2	22,25
	8	1	5,00
	Total	58	
PLT 4	1	20	26,33
	2	3	24,00
	3	13	22,58
	4	11	47,18
	5	2	39,00
	6	8	35,38
	7	2	27,50
	8	1	3,00
	Total	60	
PLT 5	1	20	26,88
	2	3	25,00
	3	13	20,92
	4	11	47,36
	5	2	46,00
	6	8	33,31
	7	2	24,50
	8	1	17,00
	Total	60	
PLT 6	1	20	24,00
	2	3	18,67

	3	12	25,00
	4	11	48,36
	5	2	41,00
	6	8	34,06
	7	2	15,50
	8	1	16,50
	Total	59	
